# Coarse-Grained Simulations of Adeno-Associated Virus and Its Receptor Reveal Influences on Membrane Lipid Organization and Curvature

**DOI:** 10.1021/acs.jpcb.4c03087

**Published:** 2024-10-02

**Authors:** Nichakorn Pipatpadungsin, Kin Chao, Sarah L. Rouse

**Affiliations:** † Department of Life Sciences, South Kensington Campus, Imperial College London, London SW7 5NH, U.K.; ‡ Department of Chemistry, Imperial College London, London W12 7TA, U.K.

## Abstract

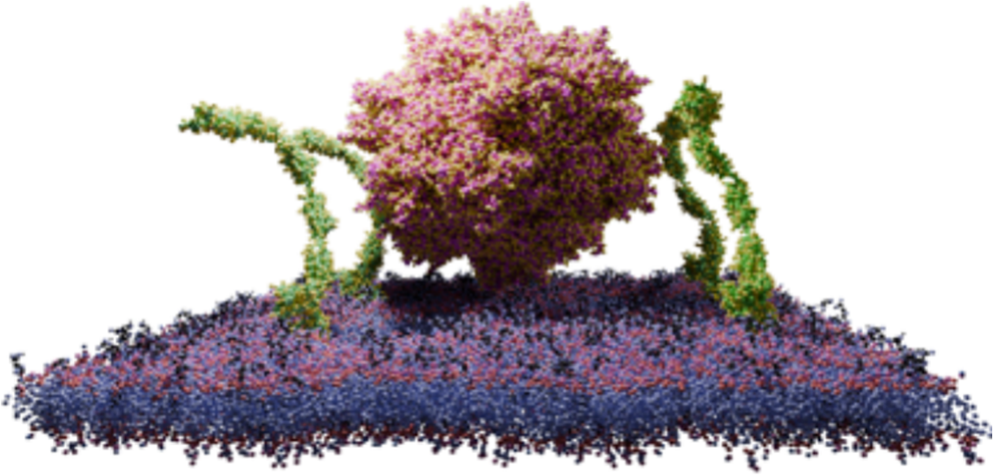

Adeno-associated virus (AAV) is a well-known gene delivery tool with a wide range of applications, including as a vector for gene therapies. However, the molecular mechanism of its cell entry remains unknown. Here, we performed coarse-grained molecular dynamics simulations of the AAV serotype 2 (AAV2) capsid and the universal AAV receptor (AAVR) in a model plasma membrane environment. Our simulations show that binding of the AAV2 capsid to the membrane induces membrane curvature, along with the recruitment and clustering of GM3 lipids around the AAV2 capsid. We also found that the AAVR binds to the AAV2 capsid at the VR-I loops using its PKD2 and PKD3 domains, whose binding poses differs from previous structural studies. These first molecular-level insights into AAV2 membrane interactions suggest a complex process during the initial phase of AAV2 capsid internalization.

## Introduction

Adeno-associated virus (AAV) is a single-stranded DNA virus belonging to the *Dependoparvovirus* genus of the family *Parvoviridae*. It was first discovered as a virus that requires adenovirus coinfection to enter a lytic phase, producing more than 100 000 particles per cell.^1^ AAV also has a high safety profile and is nonpathogenic. Hence, it has been used as a gene delivery platform with many successes in clinical trials.^2^ AAV-based vectors are already commercially available for the treatment of rare genetic disorders such as Leber’s congenital amaurosis and spinal muscular atrophy.^2^ AAV is also known for its broad and serotype-dependent tropism. However, poor tissue-specific delivery contributes to the need for high doses, causing hepatotoxicity and lethargic immune responses.^3^ Understanding the underlying biological processes of AAV infection could enable further improvements. One currently understudied area is how the AAV capsid engages with the cell’s plasma membrane. Understanding this process may elucidate useful insights into how *Dependoparvoviruses* enter the cell, which can be used for future improvement of this platform.

**Figure 1:**
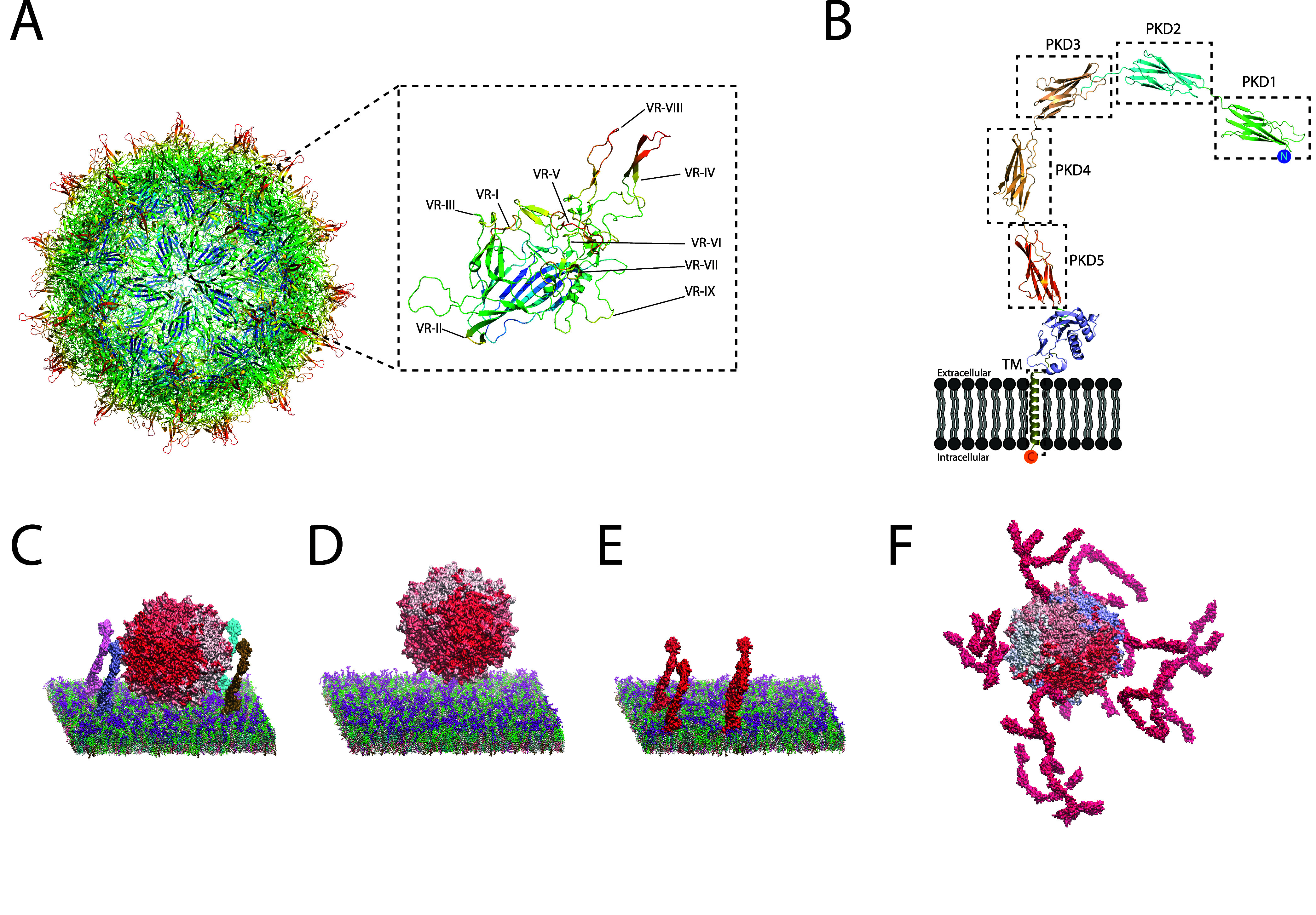
Coarse-grain modeling of the capsid and receptor systems. (A) Overview of the AAV serotype 2 capsid. The rainbow colors indicate the Euclidean distance from the center of the capsid, with red being the furthest and blue being the closest to the capsid centroid. On the right, variable region (VR) loops on a capsomer are labeled. (B) AlphaFold model of AAVR. (C) System with the AAV2 capsid and four molecules of AAVR in a realistic membrane environment. (D) Control system with the AAV2 capsid in a realistic membrane environment. (E) Control system with only four molecules of AAVR in a realistic membrane environment. (F) System of the AAV2 capsid in solution with 20 molecules of soluble (PKD1–5) AAVR.

The matured AAV virion is a single-stranded DNA encapsulated within an icosahedral capsid (triangulation number *T* = 1). Its surface features 12 fivefold symmetric pentameric faces, 20 twofold symmetric depression edges, and 20 threefold symmetric protrusions (Figure 1A). Each capsid monomer (capsomer) contains nine variable region (VR) loops, first defined by Govindasamy et al.^4^ as the region with the highest structural variations among serotypes. These loops generally form protrusions from the capsid surface (Figure 1A), including the fivefold symmetric pores at the center of each pentameric face, consisting mainly of VR-II loops, and the threefold symmetrical protrusions, where VR-IV loops show the furthest elevation from the capsid core (Figure 1A). These VR loops have been found to interact with AAV receptors and negatively charged or polar lipid heads.^5,6^ Therefore, these loops show promise in capsid engineering, with one example being a capsid mutant library that was screened to enhance delivery efficiency to the central nervous system.^7^ VR-IV has also been targeted in, for instance, nanobody insertion,^8^ HUH-tag insertion,^9^ and mCherry insertion.^10^ Meanwhile, VR-VIII has been a target for hexahistidine insertion.^11^ However, questions remain about how these modifications to the VR loops can change AAV tropism and the extent to which these modifications can be incorporated. Nevertheless, fundamentally, it is clear that VR loops play an important role in receptor usage and possess sufficient plasticity to be targeted for engineering.

In the last three decades, several cell surface receptors for the AAV capsid have been discovered. Heparan sulfate, fibroblast growth factor receptor1 (FGFR1), human growth factor receptor (HGFR), 37/67 laminin receptor, and α5β1 integrin were among the first to be discovered as the receptors for AAV serotype 2 (AAV2).^12−16^ However, FGFR1 and HGFR knocked-out haploid cells were found to be still permissible to AAV2 infection.^17^ Therefore, they may not serve as the main attachment sites. The 37/67 laminin receptor was found to be mainly expressed in the heart, skeletal muscle, and liver.^18^ Hence, this cannot explain the broad tropism of AAV2. On the other hand, α5β1 integrin is widely expressed in many tissues, serving its function as the fibronectin receptor. Hence, it is a high potential candidate for the main AAV receptor. However, results from Asokan et al.^15^ showed only one magnitude reduction in AAV2 infection when the integrin was knocked out in human embryonic kidney 293 cells. Other serotypes also seem to engage with different receptors. For instance, AAV1 and AAV6 were found to bind sialic acid, but not heparan sulfate, for cell entry.^19^ Therefore, it was presumed that different AAV serotypes may utilize different receptors for cell entry, giving rise to the variation in tissue tropism. However, genetic screening in haploid cells suggests a universal AAV receptor, AAVR.^17,20^ The focus of deciphering the AAV cell entry mechanism has therefore been shifted to the AAVR.

A motif database search with InterPro^21^ and AlphaFold structure prediction^22^ revealed that AAVR comprises an intrinsically disordered domain followed by a MANSC domain and five polycystic kidney disease (PKD) domains at its N-terminus on the extracellular side, a transmembrane (TM) helix, and an intrinsically disordered cytoplasmic C-terminal domain (Figure 1B). Many attempts have been made to determine the structure of AAVR in its AAV-bound state using mainly electron cryo-microscopy (cryo-EM) techniques. To the best of our knowledge, cryo-EM structures have been resolved with only truncated soluble extracellular domains of AAVR.^6,23−25^ In a study by Zhang et al.,^23^ the cryo-EM structure of AAV2–AAVR shows PKD2 binding with the threefold symmetric protrusions on the AAV2 capsid. Specifically, the resolved PKD2 domain was bound to VR-IV and VR-VIII on one capsomer and VR-I, VR-III, VR-V, and VR-VI on another capsomer.^23−25^ Attempts were also made to resolve the conformational flexibility of the bound AAVR molecules through electron cryo-tomography techniques.^6,26^ However, how these soluble forms of AAVR behave compared with the full-length membrane-bound form remains unknown. To date, all cryo-EM studies have resolved, at most, one domain at high resolution.^6,23−26^ This is a direct consequence of the flexibility of AAVR molecules and the symmetrization algorithm in cryo-EM reconstruction. Therefore, the conformational dynamics of AAVR and the influence of the lipid environment on capsid–AAVR binding are unknown.

In this study, we set out to explore the interaction between the AAV capsid and AAVR within the context of a model plasma membrane environment by using coarse-grained molecular dynamics simulations. Our initial focus is based on AAV serotype 2 (AAV2), which is among the best-characterized AAV serotypes.

## Methods

### Generation of the AAV2 Capsid Coarse-Grained Model

The coordinates of the atomistic model were obtained from a solved cryo-EM AAV2 VP3 capsid structure (PDB ID: 6IH9).^23^ The coordinates of five capsid monomers that form the fivefold symmetrical face in the capsid, referred to as a pentamer, were then mapped onto coarse-grained bead coordinates with the Martinize2 script.^27^ The ElNeDyn elastic network^28^ was applied to backbone beads with a force constant of 500 kJ/mol nm^2^ using a lower distance cutoff of 0 nm and an upper cutoff distance of 0.9 nm. Finally, the 12 pentamers were concatenated together into one PDB file containing 60 VP3 monomers, which make up the complete capsid, ready for building a model system (Figure S26). In this paper, we adopted a numbering system in which capsid residue 1 corresponds to residue 219 in Uniprot P03135 (Table S2).^29^

### Generation of the AAVR Coarse-Grained Model

The coordinates of the atomistic model were obtained from ColabFold.^30^ The disordered and MANSC-predicted domains at the N-terminus and the disordered cytoplasmic domain at the C-terminus were removed due to a low confidence score (Figure S27). The final coordinates contain residues 305–960 (Q8IZA0-1 Uniprot numbering).^29^ The atomistic AAVR model was oriented with respect to the plasma membrane with the N-terminus on the extracellular side using the PPM3 web server.^31^ The coordinates were mapped onto coarse-grained bead coordinates with the Martinize2 script.^27^ The ElNeDyn elastic network^28^ was applied to backbone beads with a force constant of 500 kJ/mol nm^2^ using a lower distance cutoff of 0 nm and an upper cutoff distance of 0.9 nm. In this paper, we adopted a numbering system in which AAVR residue 1 corresponds to residue 305 in Uniprot Q8IZA0-1 (Table S2).^29^

### Generation of the AAV2–AAVR Coarse-Grained Model Systems with Plasma Membrane Environment

For the system with a coarse-grained AAV2 capsid and four AAVR molecules in the plasma membrane, the coarse-grained AAV2 capsid was placed at the center of the 50 nm × 50 nm × 50 nm box, with its center ∼15 nm away from the plasma membrane. The coarse-grained AAVR molecules were randomly rotated and placed ∼2 nm away from the capsid surface, positioned along diagonals of the box in the *xy* plane (Figure 1). The coarse-grained plasma membrane model was generated using a modified Insane script,^32^ incorporating Martini3 parameters of monosialdohexosylganglioside (GM3)^33^ and phosphatidylinositol 4,5-bisphosphate (PIP2).^34^ The lipid composition of the plasma membrane was POPC/DOPC/POPE/DOPE/CHOL/DPG3 in a ratio of 25:25:8:7:25:10 in the upper leaflet and POPC/DOPC/POPE/DOPE/CHOL/POPS/DOPS/POP2 in a ratio of 5:5:20:20:25:8:7:10 in the lower leaflet. The system was then solvated with martini water beads using *gmx solvate*. Sodium (Na) and chloride (Cl) beads were added to a concentration of 0.15 M, with the system neutralized using *gmx genion*. The final number of beads of the system is summarized in Table 1. Other membrane-containing systems were generated using the same method.

### Generation of the AAV2–AAVR Coarse-Grained Model System in Solution

The coarse-grained AAV2 capsid was placed in a 50 nm × 50 nm × 50 nm box. Only the soluble PKD1–5 residues of the AAVR model (residues 305–786; Q8IZA0-1 Uniprot numbering^29^) were used. The AAVR coarse-grained models were randomly inserted 20 times using *gmx insert-molecule*, resulting in 20 molecules of AAVR in the system. The system was solvated and neutralized the same way as the membrane-containing systems. In this paper, we adopted a numbering system in which AAVR residue 1 corresponds to residue 305 in Uniprot Q8IZA0-1 (Table S2^29^).

### Molecular Dynamics

The force field used for molecular dynamics simulations is the Martini3.0 model,^35^ with the *ε* parameter in the Lennard–Jones potential between protein beads rescaled with λ_PP_ = 0.88.^36^ The standard simulation protocols from CHARMM-GUI Martini Membrane Builder were used in this study.^37^ The system was then subjected to two steps of energy minimization for 500 and 50 ps with a 0.001 ps time step, respectively, using a V-rescale thermostat and a Berendsen semi-isotropic barostat. The membrane, protein, and solute beads were assigned to three separate temperature coupling groups or to protein and solute groups in the solution system. Long-range Coulomb interactions were calculated with the reaction-field method. The Verlet cutoff scheme was used. Then, the system was subjected to five-step equilibration: (1) 1000 ps (0.002 ps time step), (2) 1000 ps (0.005 ps time step), (3) 1000 ps (0.010 ps time step), (4) 750 ps (0.015 ps time step), and (5) 10 ns (0.020 ps time step), all with the same settings as the energy minimization steps. The production run was then carried out for 10 μs (0.020 ps time step) with the same thermostat, cutoff scheme, and Coulomb potential calculation settings. The barostat in the production run was semi-isotropic Parrinello–Rahman^38^ for AAV2–AAVR, AAV2-only, and AAVR-only systems. Isotropic Parrinello–Rahman barostat^38^ was used instead in the AAV2–solution system. All systems in this study are summarized in Table S1. The temperature and pressure were set to 303.15 K and 1 bar for all steps, respectively.

Energy minimization and equilibration steps were carried out with GROMACS 2023.3.^39^ The production run was executed with GROMACS 2022.4^39^ on ARCHER2 UK National Supercomputing Service (https://www.archer2.ac.uk).

### Data Processing and Analysis

The acquired trajectories were solvent-removed with *gmx trjconv* and analyzed with in-house Python scripts based on NumPy,^40^ SciPy,^41^ Numba,^42^ MatPlotlib,^43^ Scikit-Learn,^44^ and MDAnalysis^45^ packages. All molecular model visualizations were carried out in VMD and ChimeraX.^46,47^ Unless stated otherwise, all ions and water beads were removed from the final visualizations for clarity.

#### Membrane Curvature Quantification

Membrane curvature was quantified by first fitting all PO4 beads in the upper layer of the membrane to the function
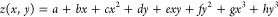
where *z*(*x*,*y*) is the fitted height (predicted *z* coordinate) of each bead as predicted by *x* and *y* coordinates. This function was selected because of its shape, which resembles the plasma membrane. The Levenberg–Marquardt algorithm, as implemented in SciPy, was used to calculate the least-squares fitting.^41^ Membrane fitting was carried out for every frame in the trajectory. The principal curvatures were calculated along the *x* and *y* axes with
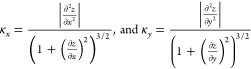
The assigned curvature for each PO4 bead was  (unsigned Gaussian curvature). Then, the average value of the unsigned Gaussian curvature () was reported for each frame.

#### Contact Analysis

A contact is defined as a phenomenon in which two reference points are closer than a 10 Å Euclidean distance. Each of the reference points is a position of the backbone (BB) bead or a position of the center of geometry for a lipid molecule. In the case of GM3 lipids, we used the carboxyl group bead (D bead) at the second sugar ring as a reference point, as its center of geometry is buried in the membrane (using nomenclature previously described^33^). The resulting contact matrices were converted to B factor columns for visualization of contact frequency (the number of contacts over time). Occupancy of lipid molecules in a residue is defined as the fraction of the number of frames in which the residue of interest is in contact with the lipid species of interest out of the total number of frames of interest.

#### Lipid Clustering Analysis

Lipid molecules were first categorized as upper-layer or lower-layer lipids based on their positions in the first frame. Lipid clustering was carried out using the Density-Based Spatial Clustering of Applications with Noise (DBSCAN) algorithm^48^ implementation in Scikit-Learn.^44^ The maximum distance for two molecules to be considered as in the neighborhood of each other was set to 28 Å, and the minimum number of elements in a cluster was set to five molecules. Reference points of the lipid molecules were referred to as their centers of geometry, and the clustering analysis was performed on these reference points for each lipid molecule type. The values for the maximum radius of a single cluster and the minimum number of elements in a single cluster were chosen to minimize the size of the nonclustered GM3 lipid group in the final frame. The frame used for optimization was from the AAV2–AAVR repeat 1 simulation.

For visualization in Figure 4A–D, the triangulated membrane depicted in 2D was colored by the average *z* coordinates of the vertices that make up each triangle. Vertices were created from a set of PO4 beads that were in the upper layer of the membrane.

#### Cross Correlation between Lipid Species and the Membrane Curvature

The membrane in each frame was first divided into a grid with each square of size 18 Å × 18 Å. Then, the cross-correlation coefficient was defined as
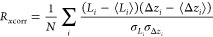
where *N* is the number of grid squares, *L*_*i*_ is the number of molecules of lipid species of interest in the grid square *i*, Δ*z*_*i*_ is the deviation of the average height (*z* value) of the PO4 beads within the grid square *i* from the average height of the PO4 beads in the entire membrane in that frame, and ⟨*X*⟩ and σ_*X*_ are the mean value and the standard deviation of variable *X* in that frame, respectively.

#### Capsid Orientation Analysis

For each repeat, we first selected two residues, with the vector pointing from one end to the other approximately parallel to the capsid diameter. We then tracked how this vector changes over time by calculating the dot product between the vector in each frame and the vector in the first frame. The output was then converted to angles in degrees.

#### GitHub Repository

All analyses described were performed with custom scripts deposited in GitHub (https://github.com/pao-pipat/CapMemUtils). Processed simulation trajectories are also available here.

## Results

### Simulations of AAV2 and AAVR in the Plasma Membrane

**Figure 2:**
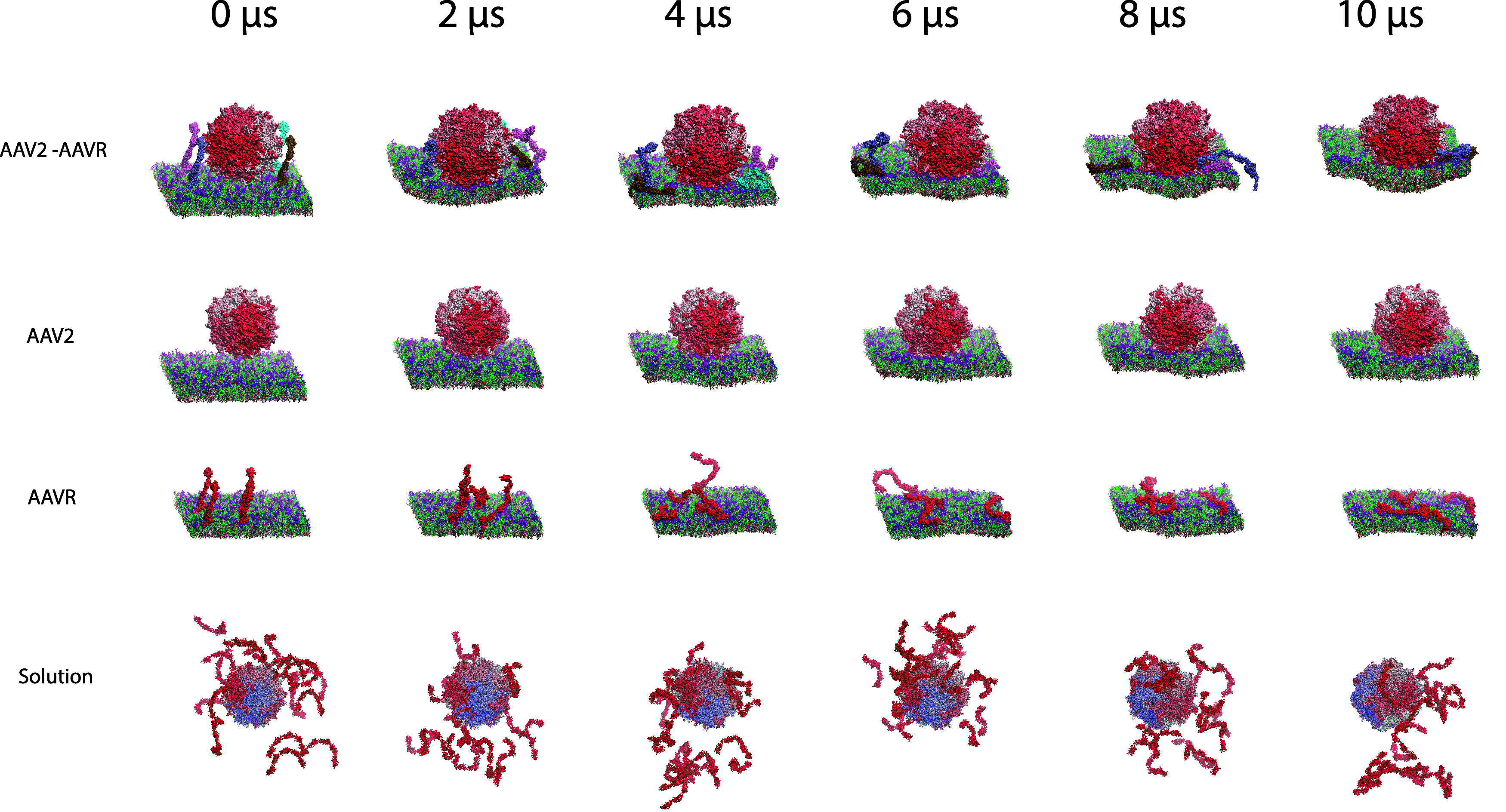
Overview of simulations. A time series showing representative systems at 2 μs intervals is shown. The repeats represented here are AAV2–AAVR repeat 1, AAV2 repeat 1, AAVR repeat 1, and solution repeat 1. Each pentamer of the capsid is colored differently from red to white to blue based on the order of residue numbers. Each receptor in the AAV2–AAVR system is colored differently. AAVR molecules in the AAVR system are colored red. Each lipid species is colored differently using VMD default colors.

To observe the initial stages of AAV2 membrane entry, we built plasma membrane models both with and without the AAVR present. Coarse-grained models of the AAV2 capsid, AAVR, and the plasma membrane were generated in a box with an edge length approximately twice the size of the capsid diameter (∼25 nm;^23^Figure 1). The systems without either the AAV2 capsid or AAVR molecules were used as controls to allow us to determine the influence of AAV2 binding on the membrane. We also constructed a system with the AAV2 capsid and 20 molecules of the AAVR soluble construct, corresponding to the conditions used in cryo-EM studies.^23^ An overview of each of these simulations at 2 μs intervals is shown in Figure 2.

We observed that AAV2 contacts the membrane within the first 4 μs of each simulation (after 2 μs for the AAV2 system or after 4 μs for the AAV2–AAVR system), allowing us to analyze the manner of binding and protein–lipid interactions in each case. The AAVR molecules are highly flexible (Figures 2 and S1). During the course of AAVR-only membrane simulations, the soluble domains of AAVR form extensive interactions with the extracellular leaflet of the membrane. This could be due to the known limitations of the Martini force field including “stickiness”^49^ but may also reflect that additional membrane components could be required to maintain the exposure of PKD domains to the extracellular environment for binding events.

To compare our membrane-containing systems simulations with the in vitro conditions that are typically used,^23^ we built a system of AAV2 and 20 molecules of soluble AAVR. AAVR molecules were observed to form loose aggregates within ∼2 μs of the simulations (Figure 2).

### AAV2 Capsid Binding to the Membrane Induces Membrane Curvature

**Figure 3:**
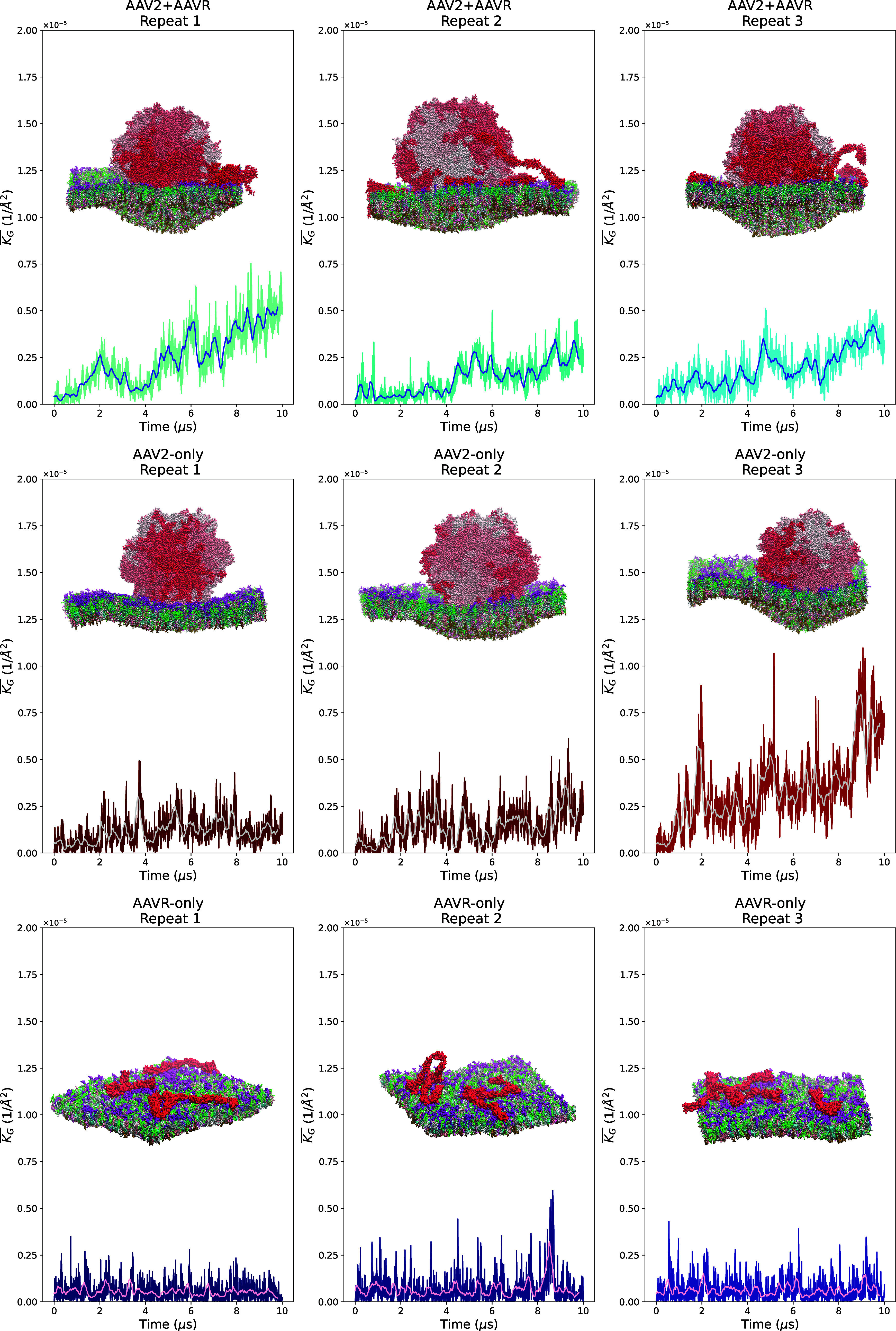
Membrane curvature plots as a function of time. The average unsigned Gaussian curvature () for each frame is represented in × 10^–5^ Å^–2^ units. Rolling average plots of 200 frames window is overlaid and colored blue for the AAV2–AAVR system, light gray for the AAV2-only system, and pink for the AAVR-only system. A snapshot of the final frame for each repeat is shown side-on to visualize the membrane curvature. Each pentamer of the capsid is colored differently from red to white based on the order of residue numbers. AAVR molecules are colored red. Each lipid species is colored differently.

One striking feature in our simulations, as shown in Figure 2, is that membrane curvature was induced after AAV2 binding. To investigate this effect further, we first modeled the membrane with a polynomial equation (see the Methods section) and derived the average unsigned Gaussian curvature, , for each frame (Figure 3).

Using this analysis, we found that after AAV2’s membrane-facing pentamers are fully in contact with the membrane (after 2 μs for the AAV2 system or after 4 μs for the AAV2–AAVR system), the curvature begins to increase (Figure 3). In the first two repeats, the AAV2 capsid on its own induced the curvature to 2.17 ± 0.16 × 10^–6^ Å^–2^ (mean ± standard error of  values in the last frame; Figure 3). However, in the third repeat, we found that the AAV2 capsid induced the curvature up to 6.91 × 10^–6^ Å^–2^ ( value from the last frame; Figure 3). For the AAV2–AAVR system, we found that the membrane curvature consistently increased toward 3.68 ± 0.51 × 10^–6^ Å^–2^ (mean ± standard error of  values in the last frame; Figure 3). Notably, the simulation of the AAV2 system third repeat was the exception, as the membrane curvature was induced dramatically after binding. This is due to the capsid membrane-facing pentamers binding to the membrane in an orientation that maximizes the contact between lipid molecules and the VR loops in the two pentamers.

Although the simulations of AAVR showed the collapse of extracellular PKD domains to the membrane (Figure 2), we found no increase in the membrane curvature (Figure 3). Therefore, the AAV2 capsid is likely the main driver of the membrane curvature. We then explored whether the presence of AAVR influences the capsid orientation to ensure that the binding interface maximizes the curvature. The orientation of the capsid relative to the first frame was measured (Figure S2). The results indicate that the AAV2–AAVR system capsid rotated more often than the AAV2-only system over 10 μs. Hence, AAVR molecules appear to influence the capsid orientation, maximizing the curvature (Figure 3). Our simulations indicate that the binding of AAVR does not directly influence the membrane curvature by itself, but its interaction with the capsid might facilitate the capsid search for the capsid orientation that maximizes the curvature.

### AAV2 Capsid Binding Causes GM3 Lipid Clustering

To understand how the AAV2 capsid binding influences the organization of lipids within the membrane, we explored the membrane lipid organization in our simulations (Figures S3–S11).

**Figure 4:**
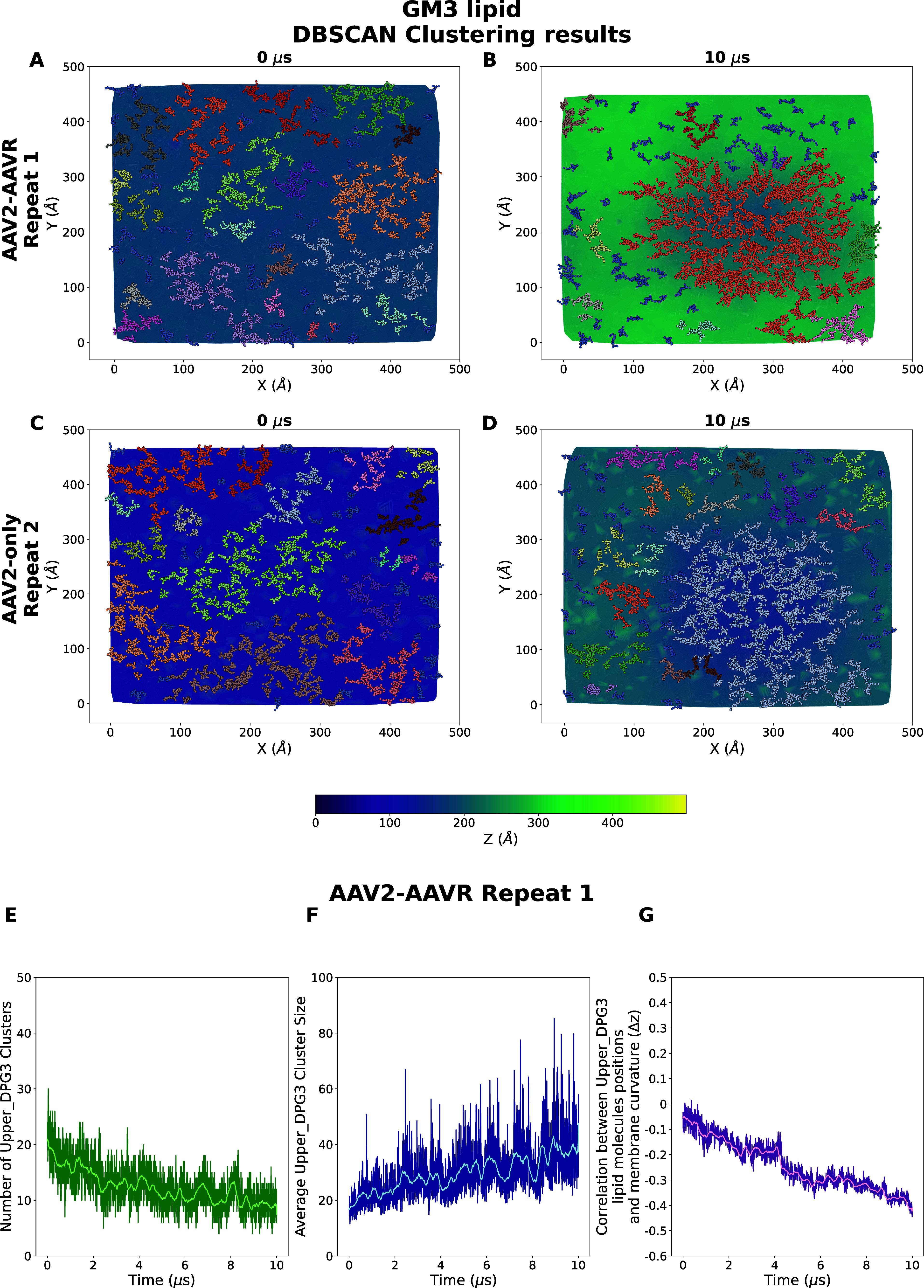

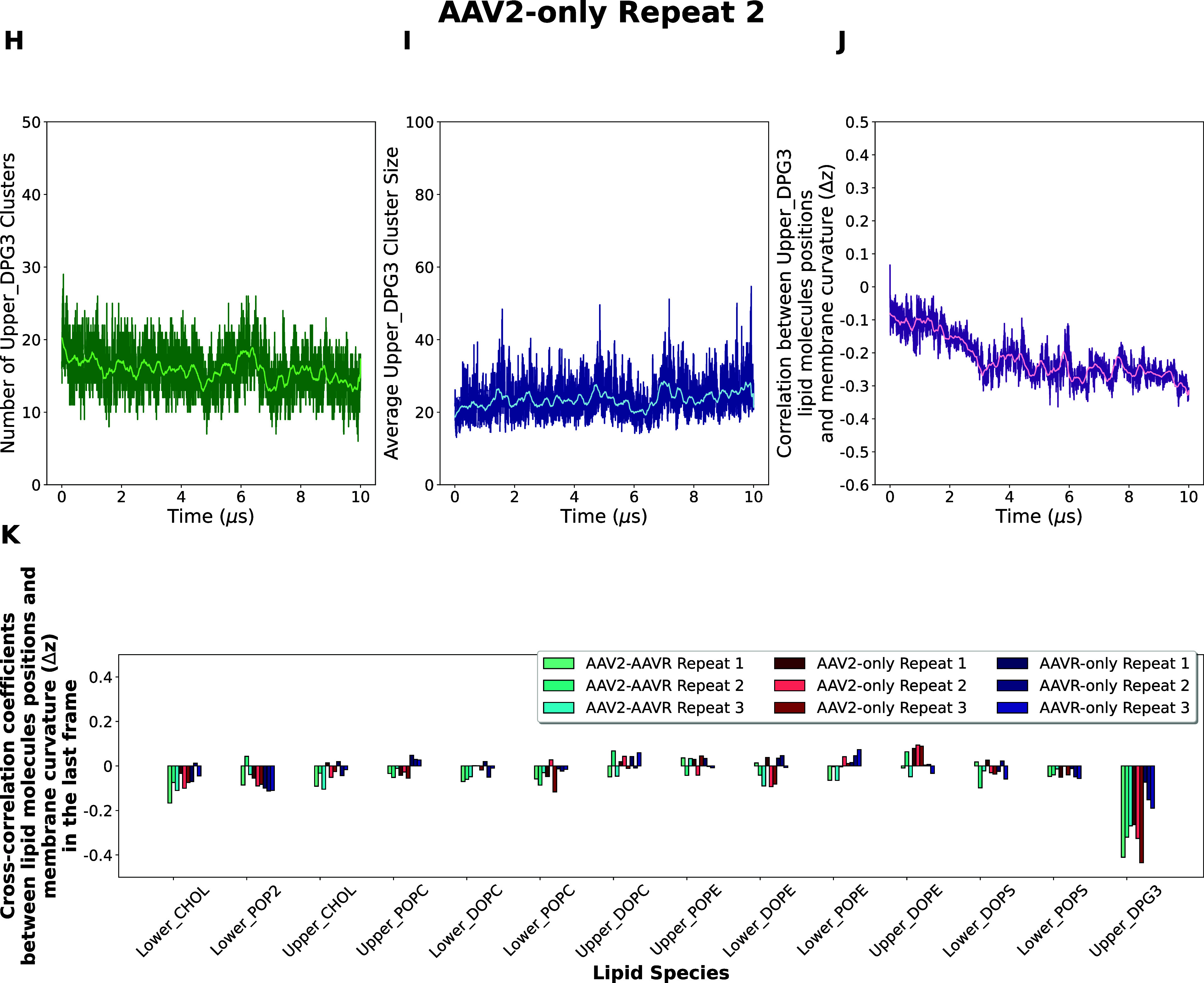
GM3 lipid clustering due to the AAV2 capsid binding. (A, B) Results from DBSCAN clustering of the AAV2–AAVR repeat 1 system at 0 (A) and 10 μs (B). (C, D) Results from DBSCAN clustering of the AAV2-only repeat 1 system at 0 (C) and 10 μs (D). The GM3 lipid molecules are colored according to their corresponding clusters. Local membrane patches are colored by the mapping of their average heights (see the color bar). (E–G) Evolutions of the number of GM3 lipid clusters (green), average GM3 lipid cluster size (blue), and the correlation between the GM3 lipid molecules positions and the membrane curvature (as represented by Δ*z*; see the Methods section) over time (pink) for AAV2–AAVR repeat 1. The rolling average window is 200 frames. (H–J) Same plots from the AAV2-only repeat 2. (K) Last frame’s local cross-correlation coefficients between the lipid molecule positions and curvature for each lipid species. Note that DPG3 and GM3 lipid names are used interchangeably. Upper and lower prefixes denote whether the lipid molecules were originally positioned in the upper or lower leaflet with Insane script, respectively.

We investigated how each lipid species correlates with the local membrane curvature, defined by the parameter Δ*z*, over time using a cross-correlation method. Δ*z* is defined as the deviation of the PO4 beads within a grid square deviate from their average position (the Methods section; Figure S12). This analysis allows us to observe whether the lipid species of interest occupy the position where the membrane is curved (Figures S3–S12). In the AAVR system, the membrane lipid species adopt an organization pattern that depends on the curvature. For example, GM3 (DPG3 beads) and PIP2 (POP2 beads) are likely to be found in negatively curved regions (convex with respect to the positive *z* direction; see Figure 4K). In the presence of the AAV2 capsid, GM3 stands out as the only lipid showing a relatively strong correlation with the negatively curved membrane (Figure 4K). The negative correlation of the GM3 lipid molecule positions with the membrane curvature also progresses over time (Figure 4G,J). Notably, the AAV2–AAVR system shows a slightly stronger negative correlation than the AAV2 system. This indicates that GM3 lipid molecules diffused with bias toward the negatively curved region where the capsid was bound.

We found that GM3 lipid molecules were organized into clusters nearby the capsid-binding site over time (Figure 4A–D), while other lipid species did not show a clear clustering (see Figures S3–S11). We calculated this by using the DBSCAN algorithm^48^ (see the Methods section). This algorithm was chosen based on its effectiveness in identifying a cluster with an arbitrary shape, which is often the case for lipid molecule clusters. Our analysis shows that the number of GM3 clusters in AAV2–AAVR and AAV2 systems decreases, while their average cluster size increases (see Figure 4E,F,H,I for representative repeats). The average cluster sizes of GM3 lipid molecules increased consistently toward ∼40 molecules per cluster in the presence of AAVR, while the average cluster sizes remained consistently near 30 molecules per cluster in the first two repeats of the AAV2 system (Figures 4F,I and S13). In the third repeat, the AAV2 system however reached a GM3 lipid cluster size of ∼40 molecules per cluster within ∼4 μs (Figure S13). The largest cluster of GM3 lipid molecules is formed in all cases within the region where the *z* coordinates of the local membrane patch are lower than the average membrane position (Figures 4B,D and S3–S11). The clustering of the system with only AAVR molecules shows no changes in the GM3 lipid cluster sizes and number (Figures S13–S14).

Taken together, our analysis shows that GM3 lipid molecule clustering near the capsid-binding site is likely an effect driven by the AAV2 capsid and modulated by the presence of AAV2–AAVR interactions.

### AAV2 Capsid’s VR Loops Are GM3 Lipid-Binding Sites

**Figure 5:**
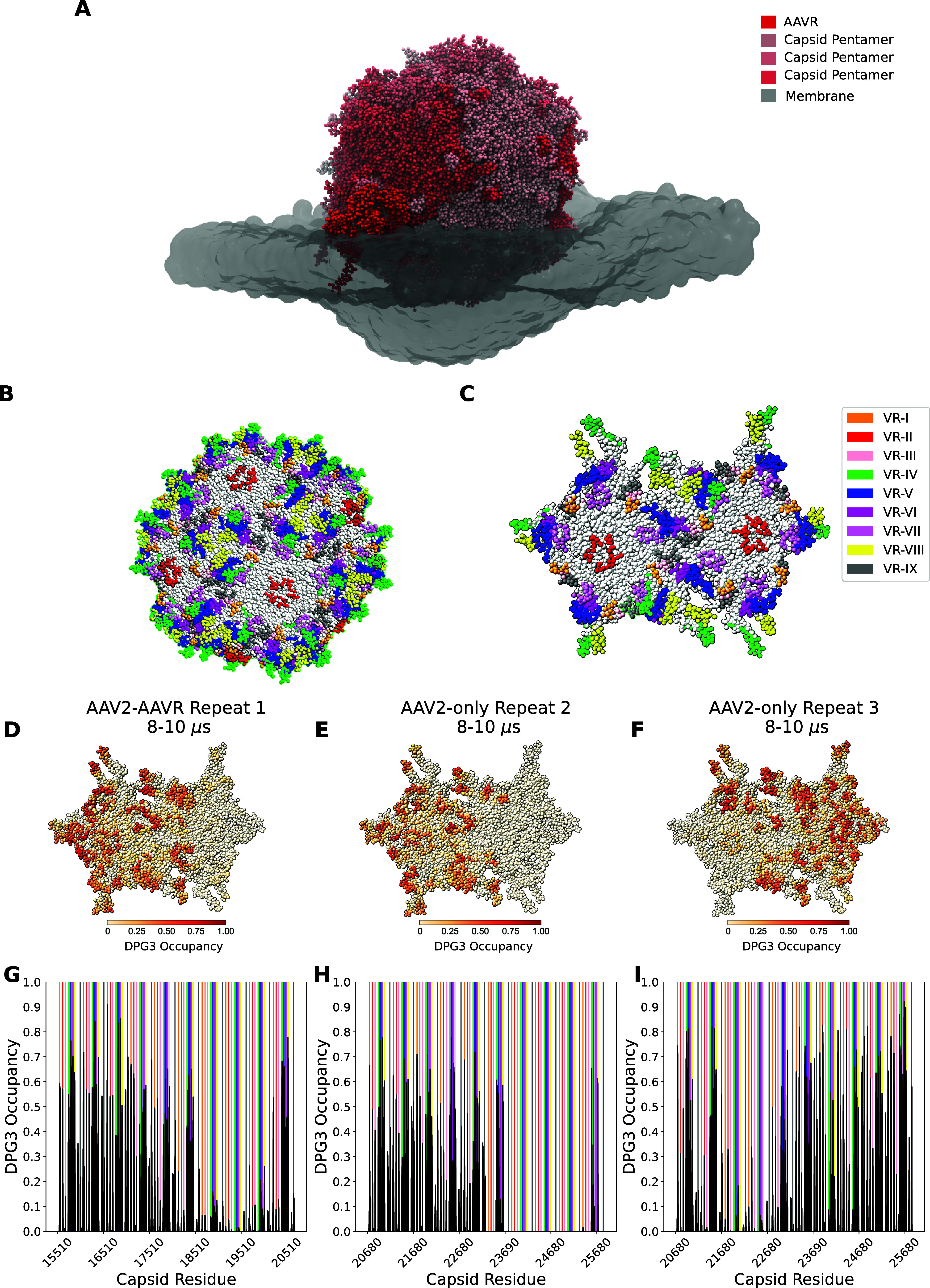
GM3 lipid-binding sites on the membrane-binding pentamers. (A) Overview of the AAV2–AAVR system in its bound state. (B) Capsid with VR loops colored as described in the legend on the right. (C) Membrane-binding pentamers with VR loops colored according to the legend on the right. (D) Membrane-binding pentamers colored by the GM3 lipid (DPG3 beads) occupancy level at 8–10 μs from the simulation of AAV2–AAVR system repeat 1. The raw occupancy score can be seen in (G) (black line). (E) Membrane-binding pentamers colored by the GM3 lipid (DPG3 beads) occupancy level at 8–10 μs from the simulation of AAV2-only system repeat 2. The raw occupancy score can be seen in (H) (black line). (F) Membrane-binding pentamers colored by the GM3 lipid (DPG3 beads) occupancy level at 8–10 μs from the simulation of AAV2-only system repeat 3. The raw occupancy score can be seen in (I) (black line).

Our simulations show that the membrane-binding face of the capsid consists of two fivefold faces (pentamers; Figures 2 and 3). We then calculated the occupancy (as defined in the Methods section) of GM3 lipid molecules on these capsid pentamers to identify the GM3-binding sites (Figure 5).

The common feature we found was that the VR-IV loops on the interface of the two pentamers correspond to the initial membrane-binding region (Figures 5, S15, and S16). Gradually, other VR loops interact with capsomers near the pentameric interface being mostly occupied by GM3 lipid molecules (Figures S15, S16, and 5). Our GM3 lipid occupancy plots (Figure 5D–I) also suggest that VR loops on the capsid were interacting with the membrane, and there is little evidence of lipid molecules bound outside VR loops. It is also apparent in our data that although all VR loops are capable of binding to GM3 lipid molecules, VR-IV loops are the most highly occupied.

Notably, during the final 2 μs of the simulations, we found two distinct binding modes of the capsid and the membrane, which led to a different final induced curvature of the membrane. Figure 5D,F, showing the binding events during the final 2 μs for AAV2–AAVR repeat 1 and AAV2 repeat 3, indicates the final orientation that maximizes the membrane curvature. The capsid appears as a V-shape so both pentameric faces are fully in contact with the membrane. On the other hand, the orientation in Figure 5E, which illustrates the binding event during the final 2 μs of the AAV2 system repeat 2 simulation, did not induce high curvature (see Figure 3), and the final occupancy shows that only one of the two membrane-binding pentamers is in contact with the GM3 lipid molecules.

Taken together, we conclude that the capsid can induce the high-curvature state of the membrane without AAVR, as shown in AAV2 system repeat 3 (Figures 3 and 5F). However, the progression toward a highly curved membrane state is ensured in the presence of AAVR, as it introduces biases in the angular orientation of the capsid (Figure S2). The effects of this can be seen from the clustering phenomenon, as shown in the previous section (Figure 4).

### VR-I Loop of the AAV2 Capsid Serves as the AAVR-Binding Site

We hypothesize, based on the analyses presented in previous sections, that AAVR binding ensures capsid interaction with the membrane (Figures 3–5). To the best of our knowledge, there have been no published structural studies on the full-length AAVR interaction with the AAV2 capsid. The structural information we have is limited to the first two PKD domains of AAVR. For instance, a structural study using single-particle cryo-EM analysis by Zhang et al.^23^ presents a structure with PKD2 bound onto the threefold protrusions (Figure 7A,C). Specifically, VR-I, VR-III, VR-V, VR-IV, and VR-VIII are present at the interface with the PKD2 domain.^23^ Nevertheless, the structure lacks dynamic information. Therefore, we have simulated a solution state system (see the Methods section) to compare our simulations with the published structures. This allows us to compare the AAVR-binding mode in the solution state with the membrane-bound form. To analyze the simulation results, we performed an occupancy analysis similar to the analysis carried out in Figure 5 (Figures S17–S25 and S28).

**Figure 6:**
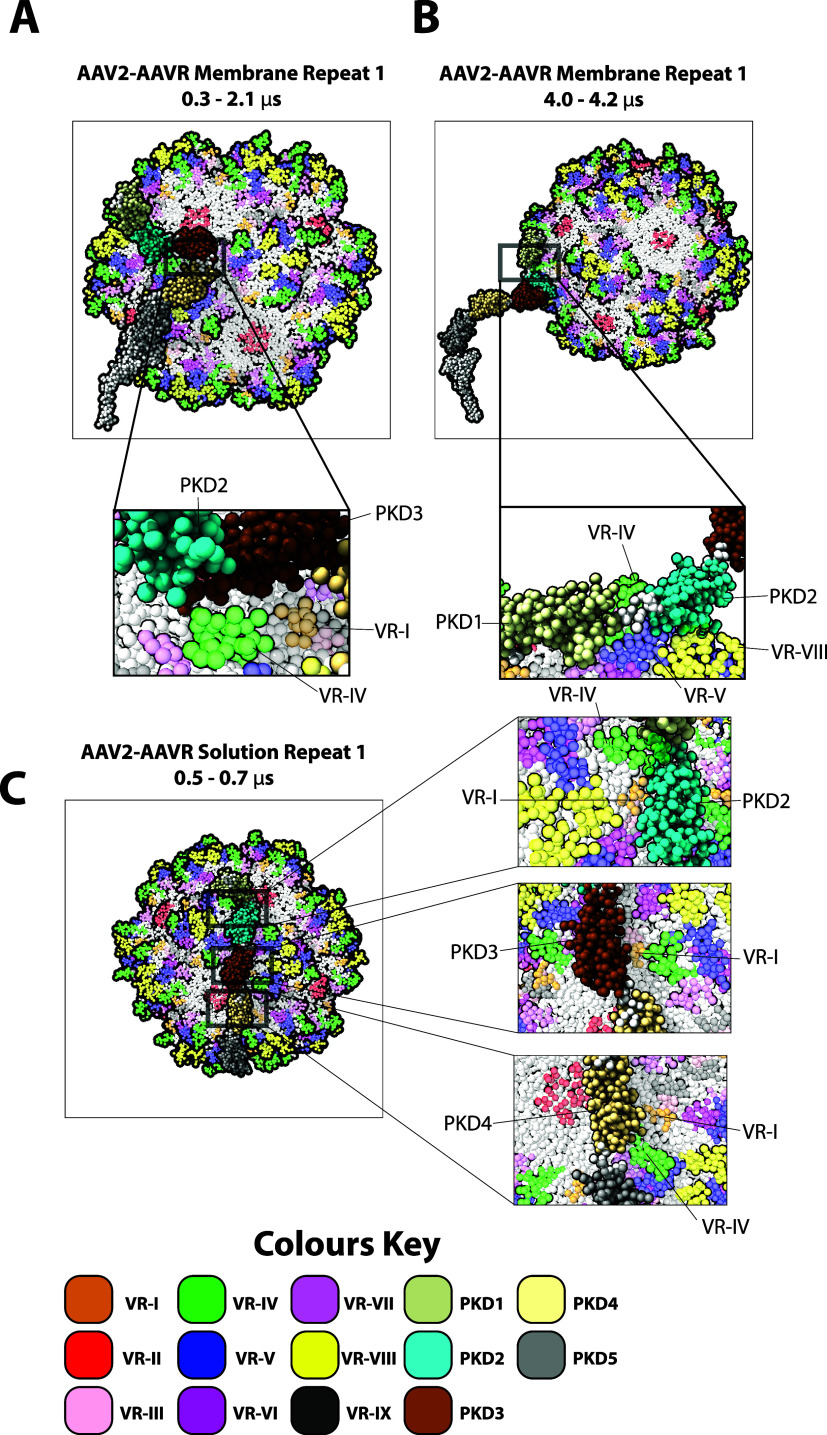
Representative subset of the AAV2–AAVR complex poses captured from AAV2–AAVR membrane system simulations, AAV2–AAVR solution system simulations, and cryo-EM. (A) AAV2–AAVR bound complex captured at 1.3 μs from the AAV2–AAVR membrane system repeat 1. (B) AAV2–AAVR bound complex captured at 4.1 μs from AAV2–AAVR membrane system repeat 1. (C) AAV2–AAVR bound complex captured at 0.6 μs from AAV2–AAVR solution system repeat 1.

**Figure 7:**
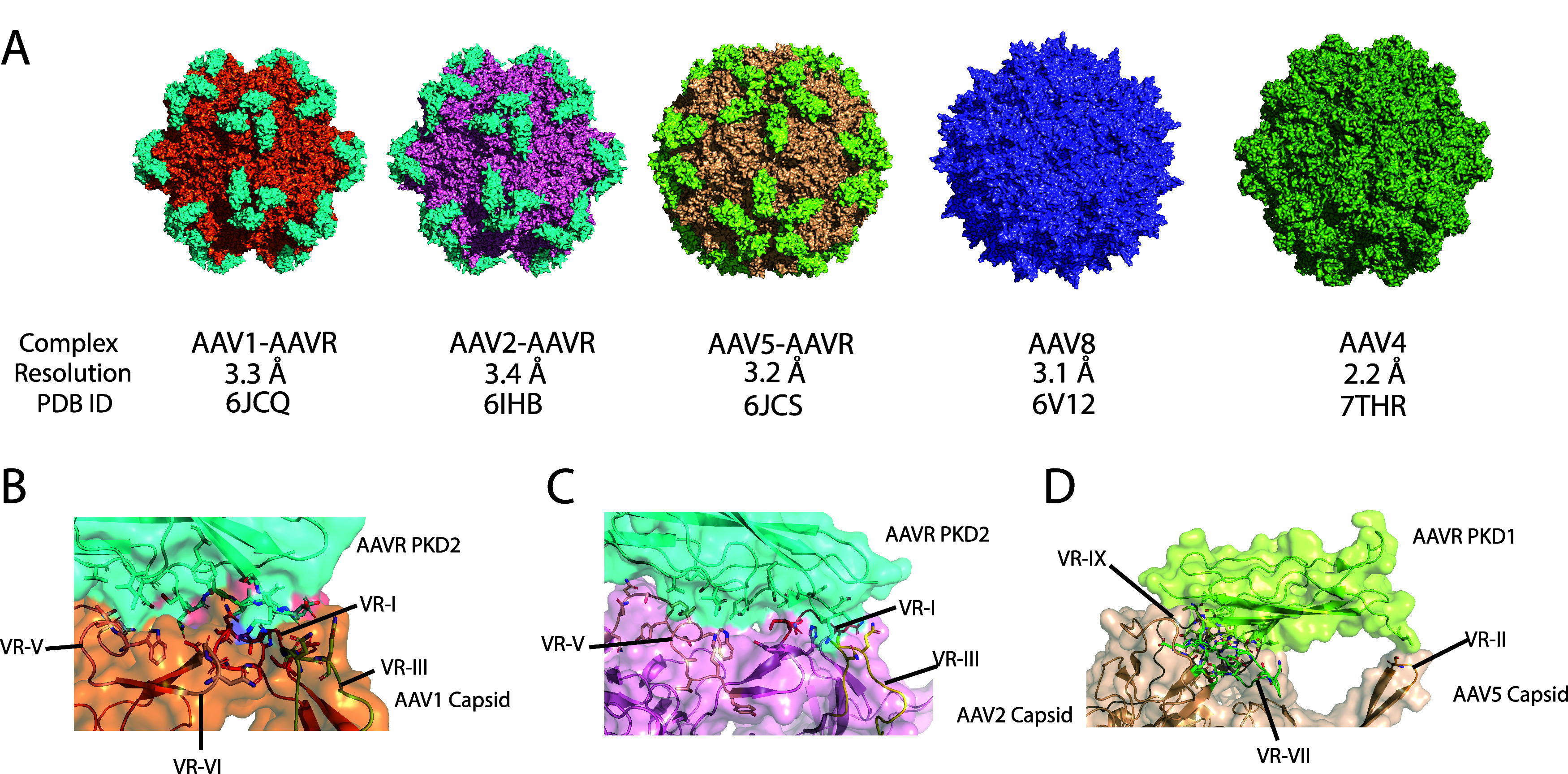
Serotype-specific AAVR-binding modes of AAV. (A) Panel showing different AAV–AAVR systems and the capsid structures that were experimentally solved by electron cryo-microscopy (PDB ID, 6JCQ from Zhang et al.;^24^ PDB ID, 6IHB from Zhang et al.;^23^ PDB ID, 6JCS from Zhang et al.;^23^ PDB ID, 6V12 from Mietzsch et al.;^52^ PDB ID, 7THR from Zane et al.^53^). (B) AAV1–AAVR closeup view. (C) AAV2–AAVR closeup view. (D) AAV5–AAVR closeup view.

Our AAV2–solution system simulations show a wide range of capsid–AAVR-binding modes (Figures 6C and S28). In Figure 6C, we observe three threefold protrusions involved with this binding. In all binding sites, residues Q45-G47 on the capsid’s VR-I loop (Figures 6C and S28) were stably bound during the binding period. AAVR’s PKD2, PKD3, and PKD4 were bound to these VR-I-binding sites. In another binding region, AAVR’s PKD4 was bound to a region near a VR-IV loop (Figure 7C). Notably, the occupancy level in this binding region is not as strong as those of the other two binding sites (Figure S28), indicating weak binding.

Our AAV2–AAVR simulations show that the AAV2 capsid first associates strongly with one of the four receptors while interacting weakly with the rest. Once the capsid approaches the membrane, interactions with the remaining AAVR occur on the membrane. Future work should include further characterization of the stoichiometry requirements of AAV with membrane-bound AAVR. We observed that the first interacting receptor always interacted with a threefold symmetric protrusion at VR-I and VR-IV loops using its PKD3 domain (Figure 7A). The first receptor also assumed another conformation in which its PKD1 and PKD2 domains moved closer to the center of a threefold protrusion near VR-IV and VR-V loops (Figure 7B). The interaction of PKD1 and PKD2 with the capsid lasted for only ∼1 μs at a time, while PKD3 stayed bound after the initial binding occurred (Figures S17–S25 and S28).

Taken together, our simulations show a diverse configurational space for AAV2–AAVR binding. A common feature that appears in all our simulations and in the cryo-EM structure is the AAVR association with the VR-I loop, especially Q45-G47 (Figures S17–S25 and S28). VR-I also appears to have no orientational preference for the interacting PKD domain. Polar–polar residue interaction between the VR-I loop and Thr, Ser, or Asn residues may stabilize this complex formation, as they show the highest occupancy score (Figures S17–S25 and S28). VR-IV and VR-V loops are also found to be another major interaction site both in our AAV2–AAVR membrane simulations and in the cryo-EM structure. However, the interaction is much weaker. We speculate that VR-I may serve as the primary docking site for AAVR. Then, the complex may be able to form in various poses due to the flexibility of the AAVR’s extracellular domains.

## Discussion

We have simulated how the AAV2 capsid engages with AAVR molecules and reorganizes the plasma membrane. Several key insights have been revealed. First, we found that the AAV2 capsid is capable of bending the membrane by itself. However, larger curvature and GM3 lipid clusters generally formed in the presence of AAVR. We also found that the capsid attracts and, hence, causes the clustering of GM3 lipid molecules within the binding region, with VR-IV being the main binding partner to GM3 lipid heads. All of these phenomena lead to a final state captured in our simulation that resembles the initial stages of AAV2 internalization.

Based on our data, we hypothesize that the formation of the initial internalization configuration is due to a positive feedback loop. The capsid first attaches to the membrane or AAVR through its VR-I loops. The capsid must then orient itself so that two of the pentameric faces of the capsid could interact with the membrane. The VR-IV loops then attach to the GM3 lipid heads, accelerating the attachment. It has also been verified experimentally with cryo-EM that VR-IV loops can interact with the polar or negatively charged lipid heads.^5^ Molecular dynamics simulations have also shown that GM3 lipid molecules can cluster in the negatively curved regions of the membrane, which is partially mediated by water and ions beads.^50^ Therefore, GM3 lipid molecules within the initial binding region likely recruit more GM3 lipid molecules into the site. The bending of the membrane also results in more surface area of interaction, resulting in a higher chance of lipid diffusion into the binding site. This creates a positive feedback loop that drives the membrane distortion, similar to what happens during an early stage of the capsid internalization process.

Our simulations suggest a wide variety of AAVR and AAV2 capsid-binding modes in the presence of the membrane compared to the previous cryo-EM solution structures. Although our simulations in solution did not capture all of the key interactions found in the literature, we found some interesting common features. Particularly, the VR-I loop is present in all binding interfaces whether in solution, model membrane environment, or experimentally solved cryo-EM structure (Figures 6 and 7). This led us to conclude that VR-I may serve as the primary binding site for AAVR. Interestingly, when Cabanes-Creus et al.^51^ engineered the VR-I loop by inserting a Thr residue between the residues corresponding to S46 and G47 in our numbering, they observed improved murine hepatocyte entry and expression in both clade B (AAV2-like capsid) and clade C (AAV3b-like capsid) AAV serotypes. The effects of this insertion also diminished in an AAVR-knockout cell line.^51^ This further suggests the importance of the VR-I loop in determining the specificity of the capsid. We also found that AAVR can interact with VR-I in many different poses, involving PKD2 and PKD3 domains in multiple orientations^23^ (Figures 6 and 7). This is possibly a limitation of our current conformational sampling capability. Future studies may investigate further by enhanced sampling methods to better define the binding conformations between the AAV2 capsid and AAVR. Overall, these simulations give the first molecular insights into membrane-bound AAVR, in which we observe the receptor adopting a membrane-interacting form within 5 μs. Further biophysical studies into the properties of membrane-bound AAVR including AAVR oligomerization states and its interaction with other membrane components are required.

It is of note that we used a force field with the Lennard–Jones potential for protein–protein interactions scaled down by a factor of 0.88 (see the Methods section).^36^ This approach has been demonstrated to prevent the overcompaction of multidomain proteins in Martini3, and in our case, it allowed AAVR to remain upright for at least 4 μs (Figures 2 and S1). It was also necessary for computational feasibility to begin with the capsid placed sufficiently close to AAVR to ensure that AAV2 and AAVR interact on the system time frame. An issue arose in the AAV2–AAVR solution system, which mimics the conditions used in the cryo-EM experiment.^23^ Despite the scaled interaction between protein beads, AAVR molecules still aggregated (Figures 2 and S1), leading to binding modes involving multiple AAVR molecules attaching to the capsid. In this work, we selected only monomeric AAVR molecules for analysis. Future studies of more complex systems may benefit from further tuning the force field.

We observed from capsid orientation analysis (Figure S2) that once the capsid was bound to the membrane, the translational and rotational degrees of freedom were limited, meaning that we would require much longer simulation times to observe full details of how the capsid may move on the membrane. Nevertheless, we observed slightly more variation in capsid orientations in the AAV2–AAVR membrane system. We interpret this as a direct consequence of AAVR binding to the capsid and ensuring an optimal pose for the capsid binding to the membrane and leading to curvature.

Our simulations did not reach further stages of the cell entry process. This is due to several factors, principally the system size limitations that are currently computationally feasible, limiting the size of the membrane patch that we could study. Our simulations used a 50 nm × 50 nm membrane, which is just enough to show the curvature of this magnitude. In fact, the highest curvature seen in our simulations during the AAV2 system’s third repeat resulted in less than half the capsid internalized.

It is also notable that we have not incorporated other reported AAV2 receptors into our simulations, except AAVR molecules. In a realistic situation, where widely expressed receptors such as α5β1 integrin and heparan sulfate proteoglycan are present, the AAV2 capsid will likely attach to those receptors as well as AAVR. The question of whether our hypothesis on the positive feedback model holds then arises. Moreover, the receptors may exist in an oligomeric state that binds to the AAV2 capsid. Future simulations may include other receptors and an oligomeric AAVR to simulate an even more complex environment.

Future studies will allow us to determine the generality of the principle of the mechanism we proposed, as previous studies have shown not only structural diversity but also variation in AAVR-binding modes and affinity among serotypes (Figure 7). It is known that AAV1 and AAV2 share the same binding mode for AAVR in solution, where the PKD2 domain interacts with the groove between protrusions in the threefold symmetric face. However, AAV5 appears to interact with PKD1 instead. AAV4 and AAV8 have a much lower affinity toward AAVR, so there are no bound structures resolved. Hence, they are less likely to use AAVR as the main driver of cell entry; however, whether the same principle of internalization process holds is a different question. Hence, future directions should include simulations with other capsid serotypes to study this process in the context of the whole *Dependoparvovirus* genus.

## Conclusions

We find that the AAV2 capsid can induce a high membrane curvature within the microsecond time frame. GM3 lipid molecules are recruited and clustered at the capsid-binding site. This clustering and membrane distortion are likely driven by a positive feedback loop that is most efficient when two membrane-facing fivefold symmetric pentameric faces make contact with the membrane. In our analysis, the capsid alone can induce this phenomenon in one of the repeats in a control AAV2 capsid-only membrane system. However, our analysis shows that AAVR molecules play a role in ensuring the correct orientation of the capsid by binding to the VR-I loops with its PKD2 and PKD3 domains, positioning two pentameric faces in contact with the membrane. We also identified the VR-IV loops as the main driver of GM3 binding and the membrane distortion phenomenon. Finally, we found that the presence of the membrane changes the orientation of AAV2–AAVR binding. Taken together, our results indicate that the dynamics of AAV2–AAVR binding are likely to be drastically different in a cellular environment from the structural studies conducted so far. We conclude that our results have shown the molecular interactions corresponding to the initial phase of AAV2 capsid attachment and internalization, and further studies are required to characterize the full mechanism of cell entry in more complex model membrane environments.

## Supporting Information Available

Tables summarizing all simulations and the residue numbering system for AAV2 capsid and AAVR models; figure illustrating an overview of simulations for every repeat and every system; figure illustrating capsid orientation analysis for AAV2–AAVR and AAV2 systems; visualization of lipid distribution on the membrane by species for AAV2–AAVR repeat 1, AAV2–AAVR repeat 2, AAV2–AAVR repeat 3, AAV2 repeat 1, AAV2 repeat 2, and AAV2 repeat 3; visualization of lipid distribution on the membrane by species for AAVR repeat 1, AAVR repeat 2, and AAVR repeat 3; figure illustrating cross correlation between lipid species position and membrane curvature for all lipid species in all systems and repeats; figures illustrating cluster sizes and number of clusters for all lipid species in all systems and repeats; figures illustrating occupancy plots and diagrams of GM3 lipid molecules on the membrane-binding pentamers in the AAV2–AAVR system; figures illustrating occupancy of AAVR3 on the membrane-binding pentamers in AAV2–AAVR system repeat 1, AAVR4 on the membrane-binding pentamers in AAV2–AAVR system repeat 2, and AAVR2 on the membrane-binding pentamers in AAV2–AAVR system repeat 3; figures illustrating occupancy of AAVR molecules on the capsid in the solution system repeat 1, solution system repeat 2, and solution system repeat 3 sampled at every 2 μs; figures illustrating occupancy of the capsid on all AAVR molecules in the solution system repeat 1, solution system repeat 2, and solution system repeat 3 sampled at every 2 μs; figure illustrating how the AAV2 capsid and AAVR coarse-grained models were built; and figure showing a representative subset of AAV2–AAVR complex poses and its occupancy scores (PDF)
