# Supplementary material for: Coarse-Grained Simulations of Adeno-Associated Virus and Its Receptor Reveal Influences on Membrane Lipid Organization and Curvature
Source: J Phys Chem B. 2024 Oct 2;128(41):10139–53. doi: 10.1021/acs.jpcb.4c03087 (PMC11492248; doi:10.1021/acs.jpcb.4c03087)
Supplement: Supplementary file 1 — jp4c03087_si_001.pdf [file jp4c03087_si_001.pdf]

Supplementary Information: Coarse-grained simulations of adeno-associated virus and its receptor reveal influences on membrane lipid organisation and curvature.

Nichakorn Pipatpadungsin[a], Kin Chao[b], Sarah Rouse[a*]

[a] Department of Life Sciences, South Kensington Campus, Imperial College London, London, SW7 5NH, United Kingdom

[b] Department of Chemistry, Imperial College London, London, W12 7TA, United Kingdom.

[*] Email: s.rouse@imperial.ac.uk

Supplementary Information

Table S1 Summary of all simulations

Name	*System Composition*	*Total Simulated time (μs)*	*Total number of beads in simulation*	*Total Number of Repeats*	*Box dimension (nm x nm x nm)*
AAV2-AAVR	AAV2 + 4xAAVR + Membrane	10	920004	3	50 x 50 x50
AAV2	AAV2 + Membrane	10	919135	3	50 x 50 x50
AAVR	AAVR + Membrane	10	881987	3	50 x 50 x50
Solution	AAV2 + 20xAAVR	10	892754	3	50 x 50 x50

Table S2 Summary of all sequences used

Sequence	No. of Copies within a system	System
Residues: 1-517 (Our Numbering) or 219-735 (P03135 Uniprot Numbering)<br>Full Sequence:<br>DGVGNSSGNWHCDSTWMGDRVITTSTRTWALPTYNNH<br>LYKQISSQSGASNDNHYFGYSTPWGYFDFNRFHCHFSP<br>RDWQRLINNNWGFRPKRLNFKLFNIQVKEVTQNDGTTTI<br>ANNLTSTVQVFTDSEYQLPYVLGSAHQGCLPPFPADVF<br>MVPQYGYLTLNNGSQAVGRSSFYCLEYFPSQMLRTGNN<br>FTFSYTFEDVPFHSSYAHSQSLDRLMNPLIDQYLYYLSRT<br>NTPSGTTTQSRLQFSQAGASDIRDQSRNWLPGPCYRQ<br>QRVSKTSADNNNSEYSWTGATKYHLNGRDSLVNPGPA<br>MASHKDDEEKFFPQSGVLIFGKQGSEKTNVDIEKVMITD<br>EEEIRTTNPVATEQYGSVSTNLQRGNRQAATADVNTQG<br>VLPGMVWQDRDVYLQGPIWAKIPHTDGHFHPSPLMGG<br>FGLKHPPPQILIKNTPVPANPSTTFSAAKFASFITQYSTGQ<br>VSVEIEWELQKENSKRWNPEIQYTSNYNKSVNVDFTVD<br>TNGVYSEPRPIGTRYLTRNL	60	AAV2-AAVR (Membrane)<br>AAV2-only (Membrane)<br>AAV2-AAVR (Solution)
Residues: 1-656 (Our numbering) or 305-960 (Q8IZA0-1 Uniprot Numbering)<br>Full Sequence:<br>IKELVVSAGESVQITLPKNEVQLNAYVLQEPPKGETY<br>TYDWQLITHPRDYSGEMEGKHSQILKLSKLTPGLYE<br>FKVIVEGQNAHGEGYVNVTVKPEPRKNRPPIAIVSP<br>QFQEISLPTTSTVIDGSQSTDDDKIVQYHWEELKGP<br>LREEKISEDTAILKLSKLVPGNYTFSLTVVDSDGATN<br>STTANLTVNKAVDYPPVANAGPNQVITLPQNSITLFG<br>NQSTDDHGITSYEWSLSPSSKGKVVEMQGVRTPTL<br>QLSAMQEGDYTYQLTVTDTIGQQATAQVTVIVQPEN<br>NKPPQADAGPDKELTLPVDSTTLDGSKSSDDQKIIS	4	AAV2-AAVR (Membrane)<br>AAVR-only (Membrane)

YLWEKTQGPDGVQLENANSSVATVTGLQVGTYVFT<br>LTVKDERNLQSQSSVNVIVKEEINKPPIAKITGNVVIT<br>LPTSTAELDGSKSSDDKGIVSYLWTRDEGSPAAGE<br>VLNHSDHHPILFLSNLVEGTYTFHLKVTDAKGESDT<br>DRTTVEVKPDPRKNNLVEIILDINVSQLTERLKGMFI<br>RQIGVLLGVLDSDIIVQKIQPYTEQSTKMVFFVQNEP<br>PHQIFKGHEVAAMLKSELRKQKADFLIFRALEVNTV<br>TCQLNCSDHGHCDSFTKRCICDPFWMENFIKVQLR<br>DGDSNCEWSVLYVIIATFVIVVALGILSWTVICCCKR<br>QKGK		
Residues: 1-482 (Our numbering) or 305-786 (Q8IZA0-1 Uniprot Numbering)<br>Full Sequence:<br>IKELVVSAGESVQITLPKNEVQLNAYVLQEPPKGETY<br>TYDWQLITHPRDYSGEMEGKHSQILKLSKLTPGLYE<br>FKVIVEGQNAHGEGYVNVTVKPEPRKNRPPIAIVSP<br>QFQEISLPTTSTVIDGSQSTDDDKIVQYHWEELKGP<br>LREEKISEDTAILKLSKLVPGNYTFSLTVVDSDGATN<br>STTANLTVNKAVDYPPVANAGPNQVITLPQNSITLFG<br>NQSTDDHGITSYEWSLSPSSKGKVVEMQGVRTPTL<br>QLSAMQEGDYTYQLTVTDTIGQQATAQVTVIVQPEN<br>NKPPQADAGPDKELTLPVDSTTLDGSKSSDDQKIIS<br>YLWEKTQGPDGVQLENANSSVATVTGLQVGTYVFT<br>LTVKDERNLQSQSSVNVIVKEEINKPPIAKITGNVVIT<br>LPTSTAELDGSKSSDDKGIVSYLWTRDEGSPAAGE<br>VLNHSDHHPILFLSNLVEGTYTFHLKVTDAKGESDT<br>DRTTVEVKPDPR	20	AAV2-AAVR (Solution)

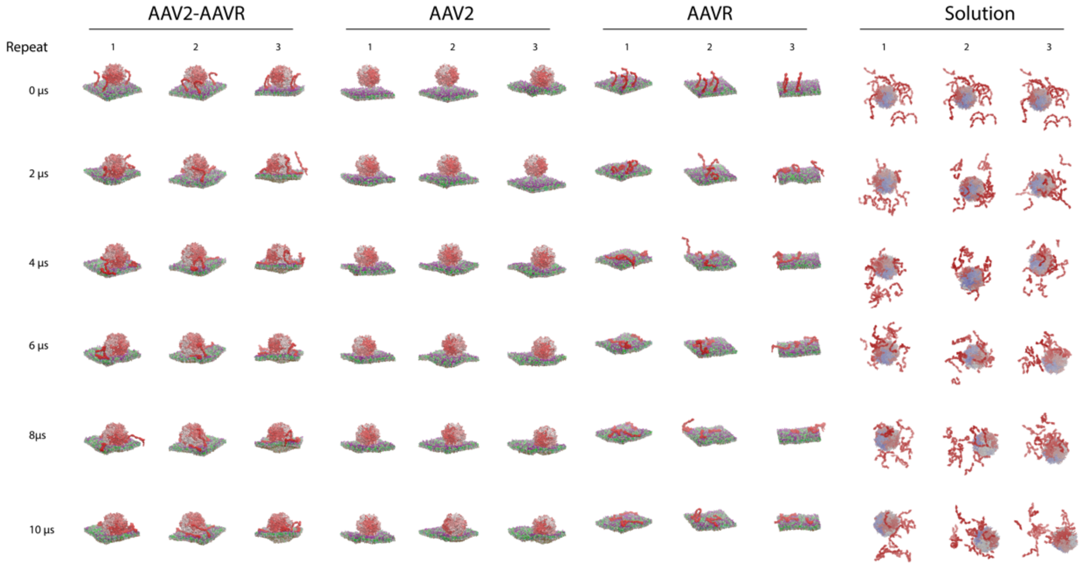

Figure S1 Overview of simulations. *A time series showing each system at 2 µs intervals is shown. Each pentamer of the capsid is coloured differently from red to white to blue based on the order of residue numbers. AAVR molecules are coloured red. Each lipid species is coloured differently.*

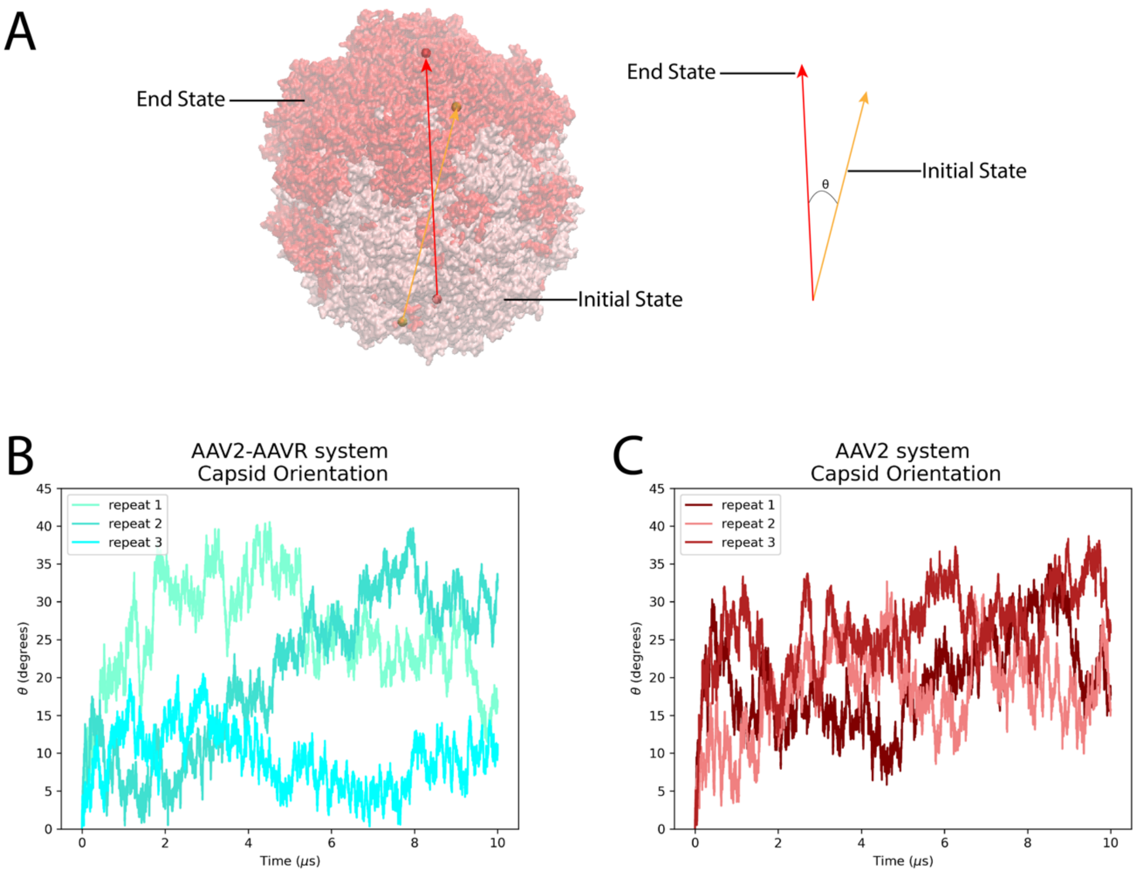

Figure S2 Capsid Orientation Analysis* | *A *On the left is an overlay between the capsid initial state (first frame; pink) and its final state (last frame; red). For the initial states the BB beads of the residues used to construct the antipodal vector are labelled in yellow. The same vector in the last frame and its residue positions are labelled in red. On the right is a not-to-scale illustration of the analysis. θ is the angle between the vector in its initial state and the final state. We performed the same analysis for this vector in every frame (see Methods).* | ***B and C*** *The results from analysing the AAV2 capsid in AAV2-AAVR system simulations and the AAV2 capsid in AAV2 system simulations, respectively.*

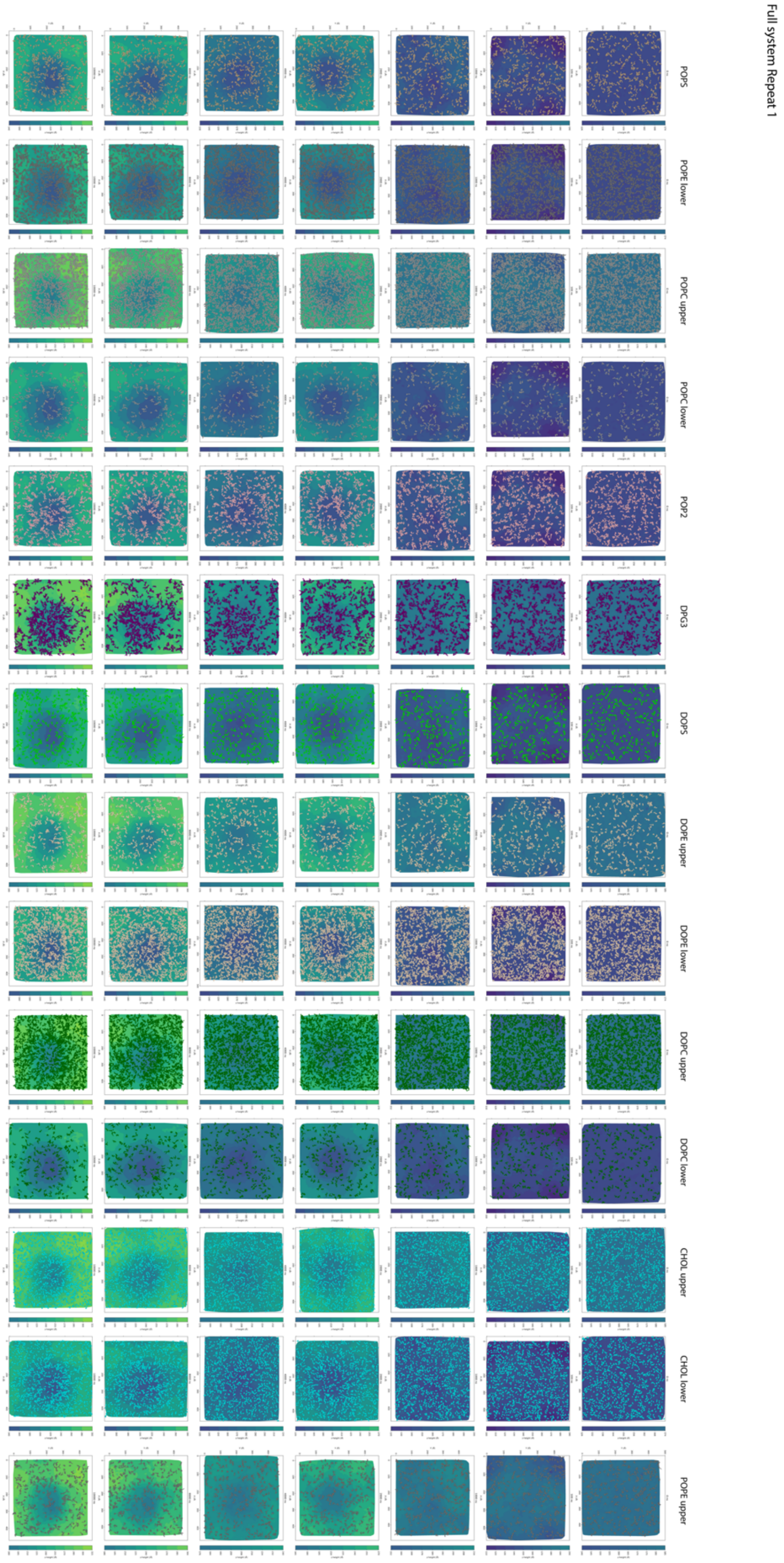

Figure S3 Visualisation of the distribution of membrane lipid species in AAV2-AAVR system repeat 1 | *In each plot the membrane leaflet surface was plotted based on the average z-height of a triangulated region created by 3 neighbouring PO4 beads. The lipid molecules are coloured differently to visualise their positions. Z-heights colour map is shown in the colour bars.*

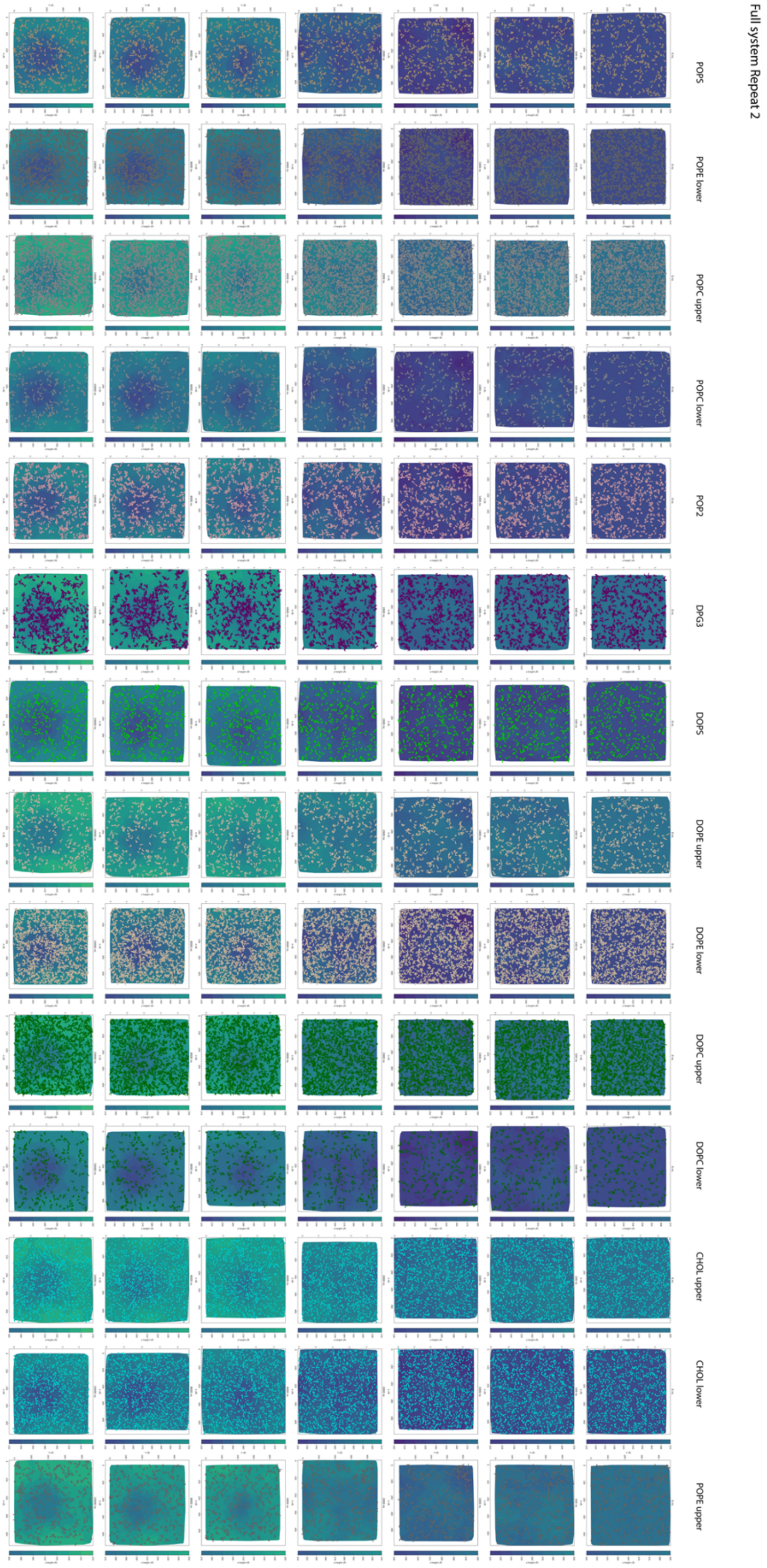

Figure S4 Visualisation of the distribution of membrane lipid species in AAV2-AAVR system repeat 2 | *In each plot the membrane leaflet surface was plotted based on the average z-height of a triangulated region created by 3 neighbouring PO4 beads. The lipid molecules are coloured differently to visualise their positions. Z-heights colour map is shown in the colour bars.*

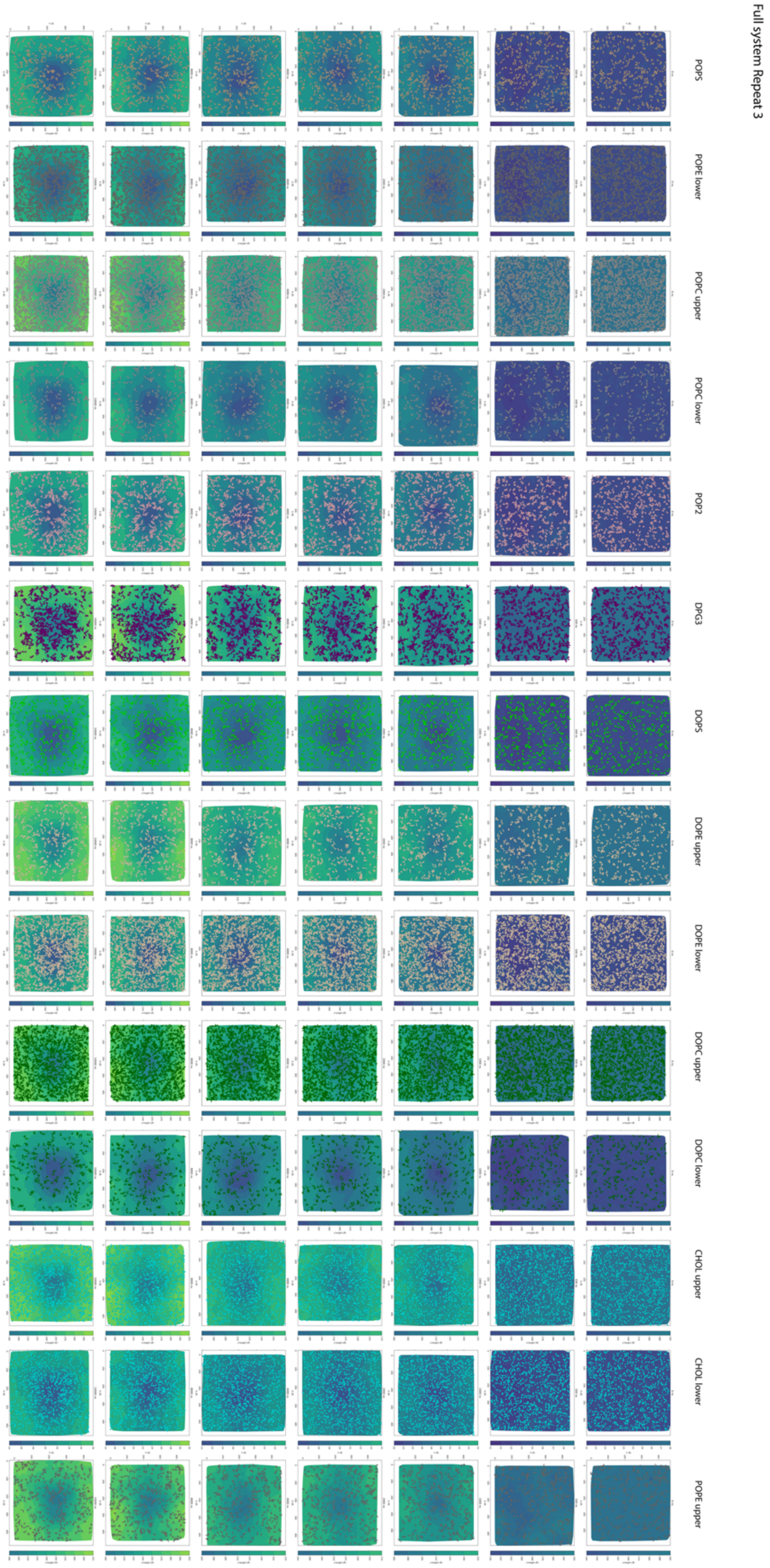

Figure S5 Visualisation of the distribution of membrane lipid species in AAV2-AAVR system repeat 3 | *In each plot the membrane leaflet surface was plotted based on the average z-height of a triangulated region created by 3 neighbouring PO4 beads. The lipid molecules are coloured differently to visualise their positions. Z-heights colour map is shown in the colour bars.*

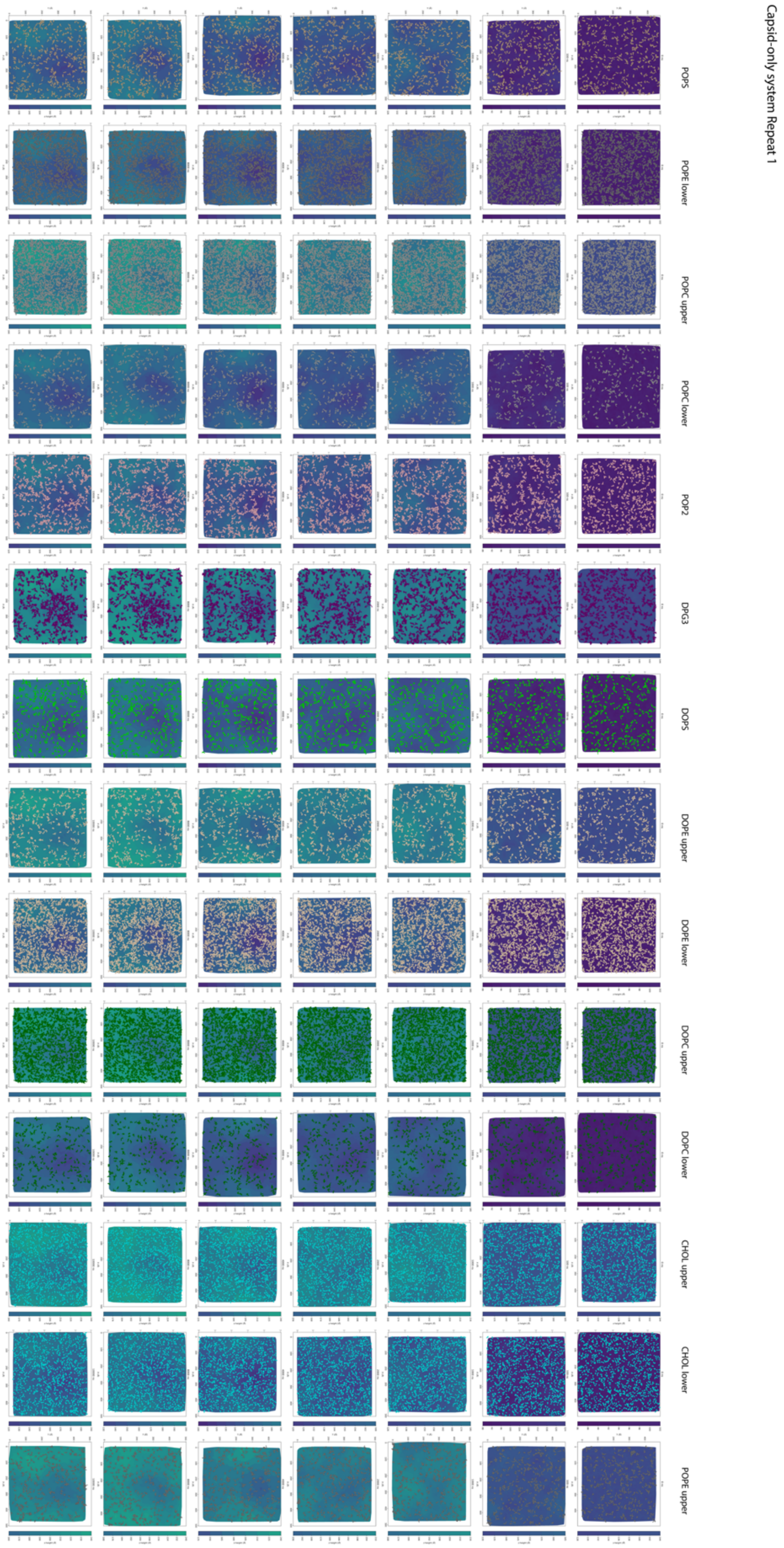

Figure S6 Visualisation of the distribution of membrane lipid species in AAV2 system repeat 1 | *In each plot the membrane leaflet surface was plotted based on the average z-height of a triangulated region created by 3 neighbouring PO4 beads. The lipid molecules are coloured differently to visualise their positions. Z-heights colour map is shown in the colour bars.*

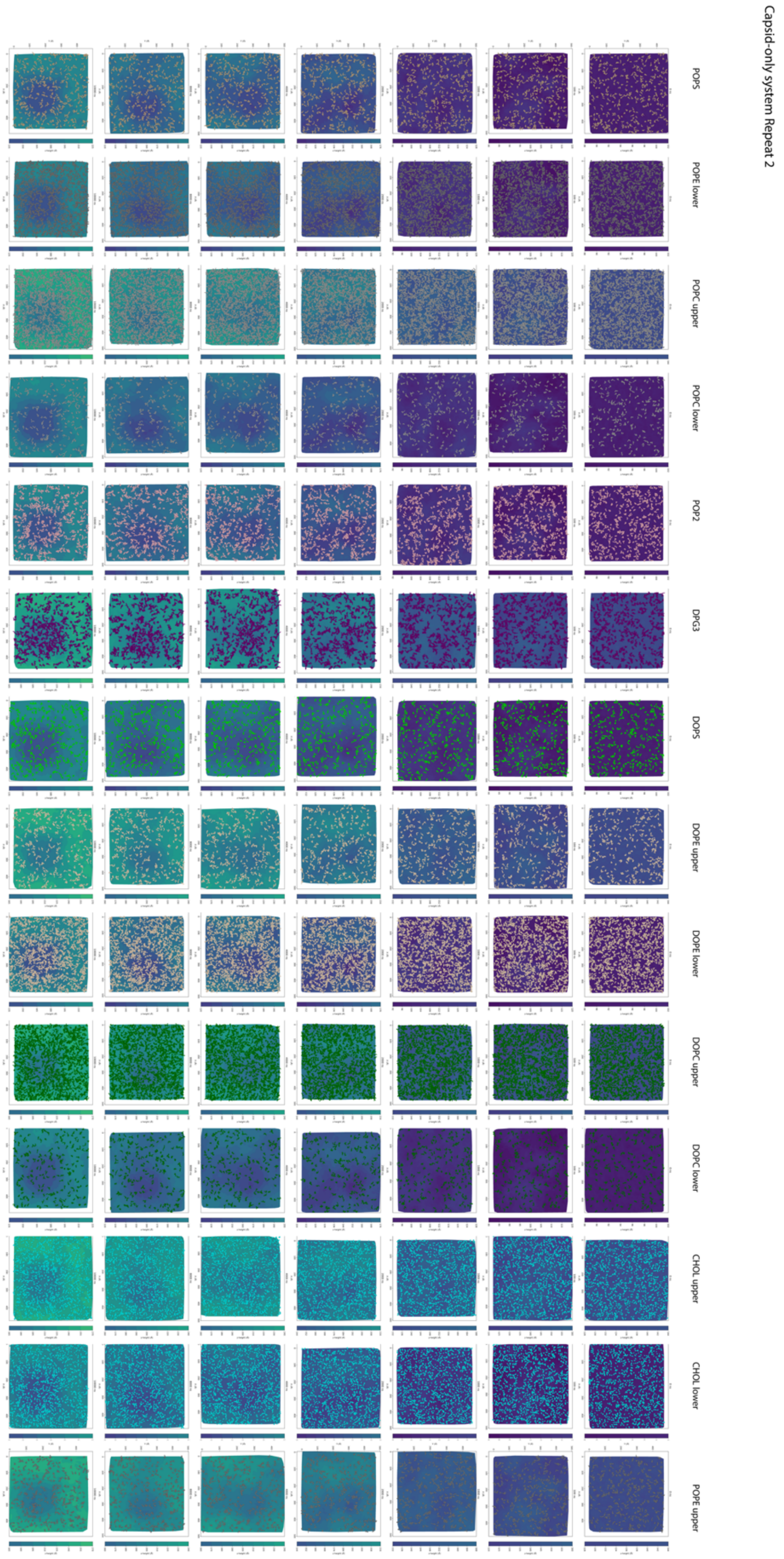

Figure S7 Visualisation of the distribution of membrane lipid species in AAV2 system repeat 2 | *In each plot the membrane leaflet surface was plotted based on the average z-height of a triangulated region created by 3 neighbouring PO4 beads. The lipid molecules are coloured differently to visualise their positions. Z-heights colour map is shown in the colour bars.*

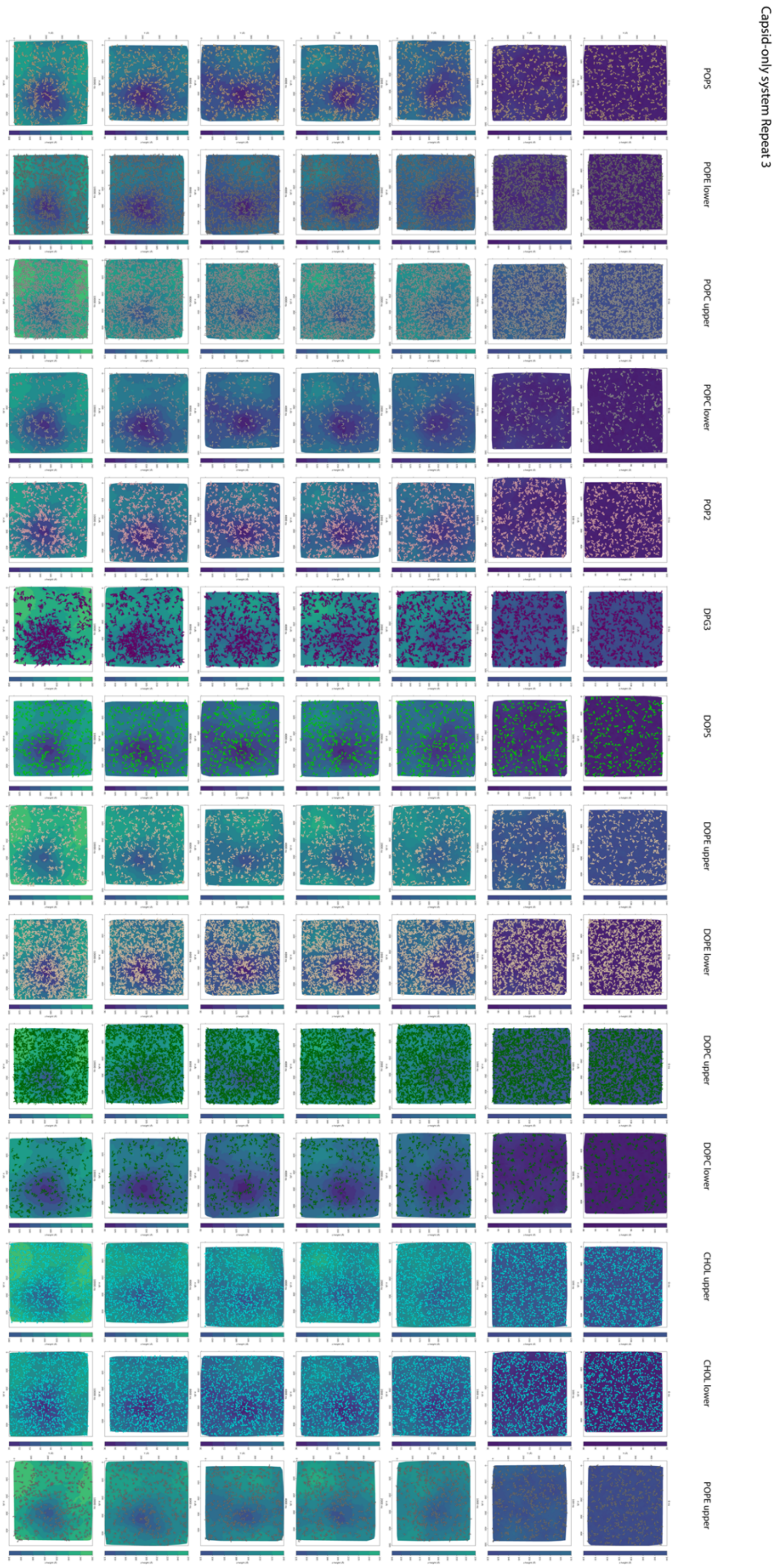

Figure S8 Visualisation of the distribution of membrane lipid species in AAV2 system repeat 3 | *In each plot the membrane leaflet surface was plotted based on the average z-height of a triangulated region created by 3 neighbouring PO4 beads. The lipid molecules are coloured differently to visualise their positions. Z-heights colour map is shown in the colour bars.*

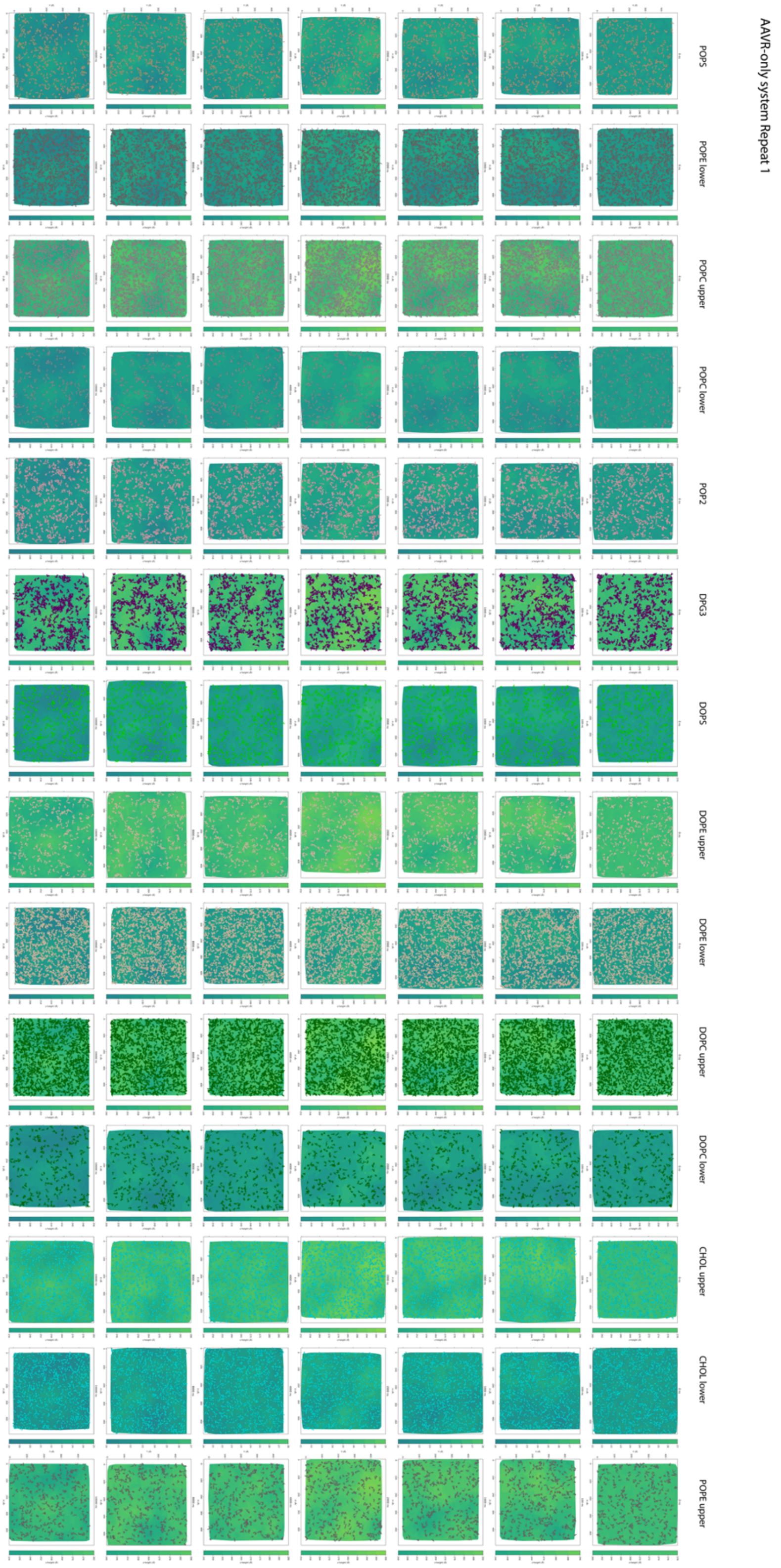

Figure S9 Visualisation of the distribution of membrane lipid species in AAVR system repeat 1 | *In each plot the membrane leaflet surface was plotted based on the average z-height of a triangulated region created by 3 neighbouring PO4 beads. The lipid molecules are coloured differently to visualise their positions. Z-heights colour map is shown in the colour bars.*

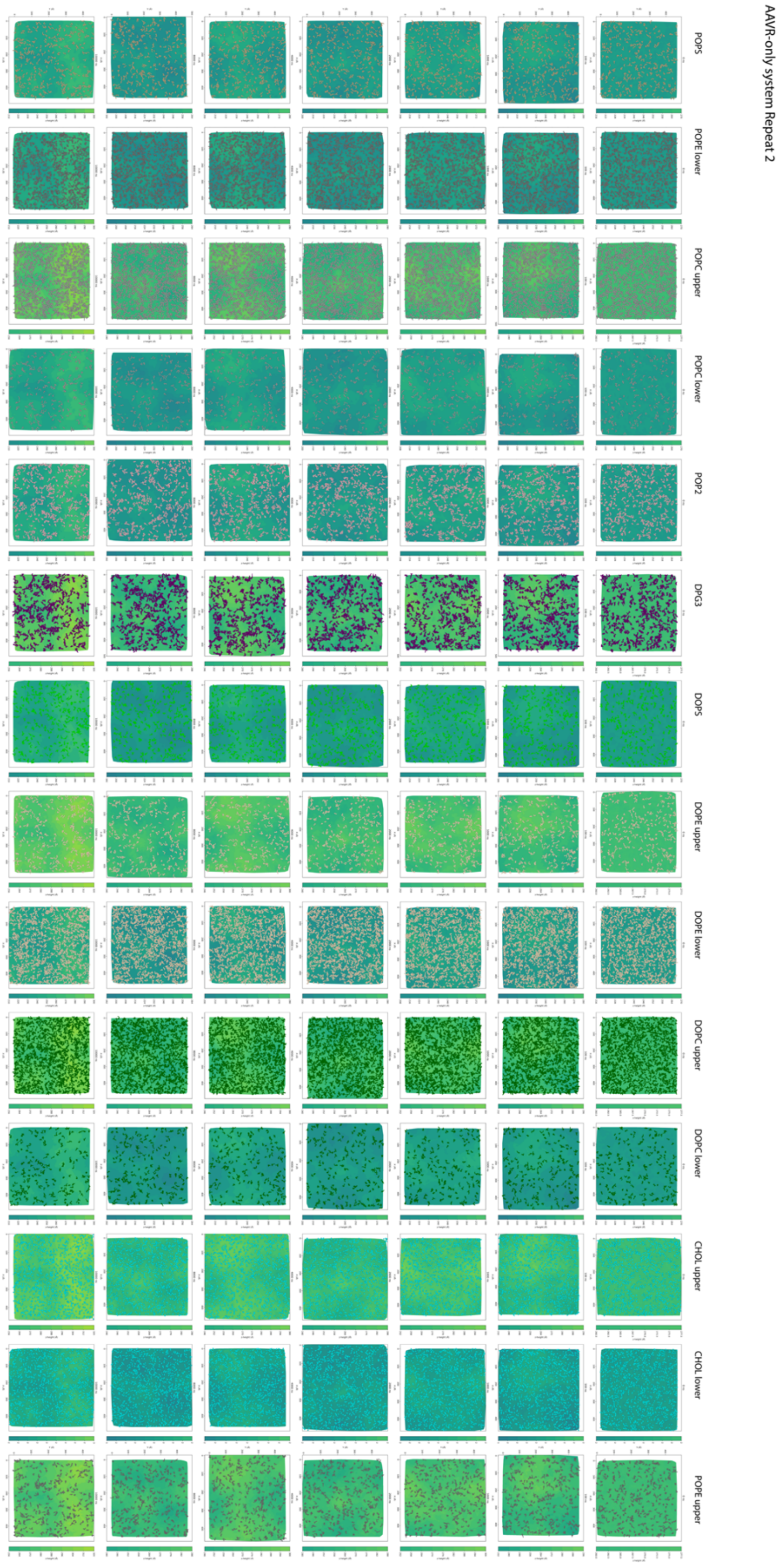

***Figure S10 Visualisation of the distribution of membrane lipid species in AAVR system repeat 2 |** In each plot the membrane leaflet surface was plotted based on the average z-height of a triangulated region created by 3 neighbouring PO4 beads. The lipid molecules are coloured differently to visualise their positions. Z-heights colour map is shown in the colour bars.*

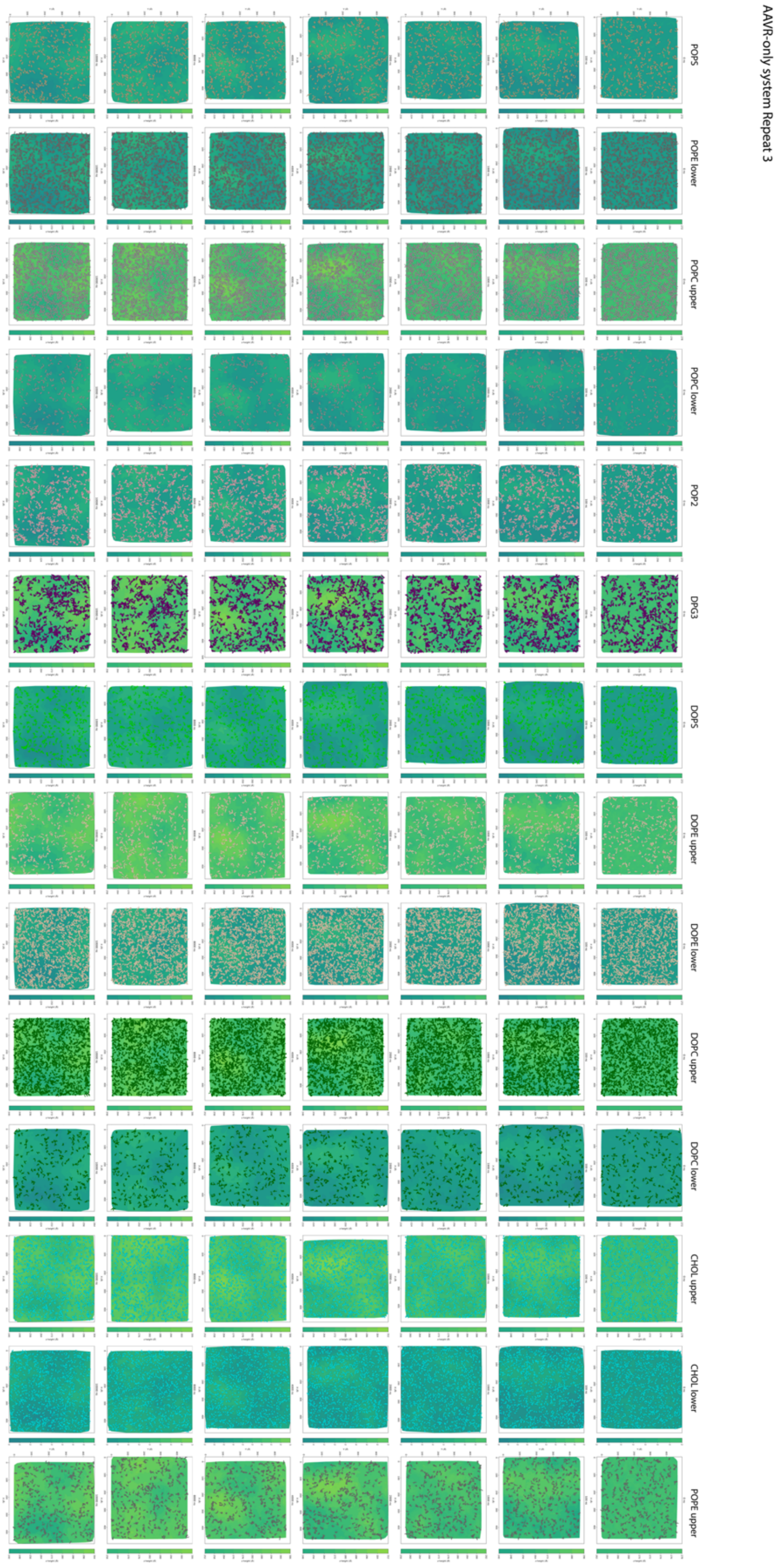

Figure S11 Visualisation of the distribution of membrane lipid species in AAVR system repeat 3 | *In each plot the membrane leaflet surface was plotted based on the average z-height of a triangulated region created by 3 neighbouring PO4 beads. The lipid molecules are coloured differently to visualise their positions. Z-heights colour map is shown in the colour bars.*

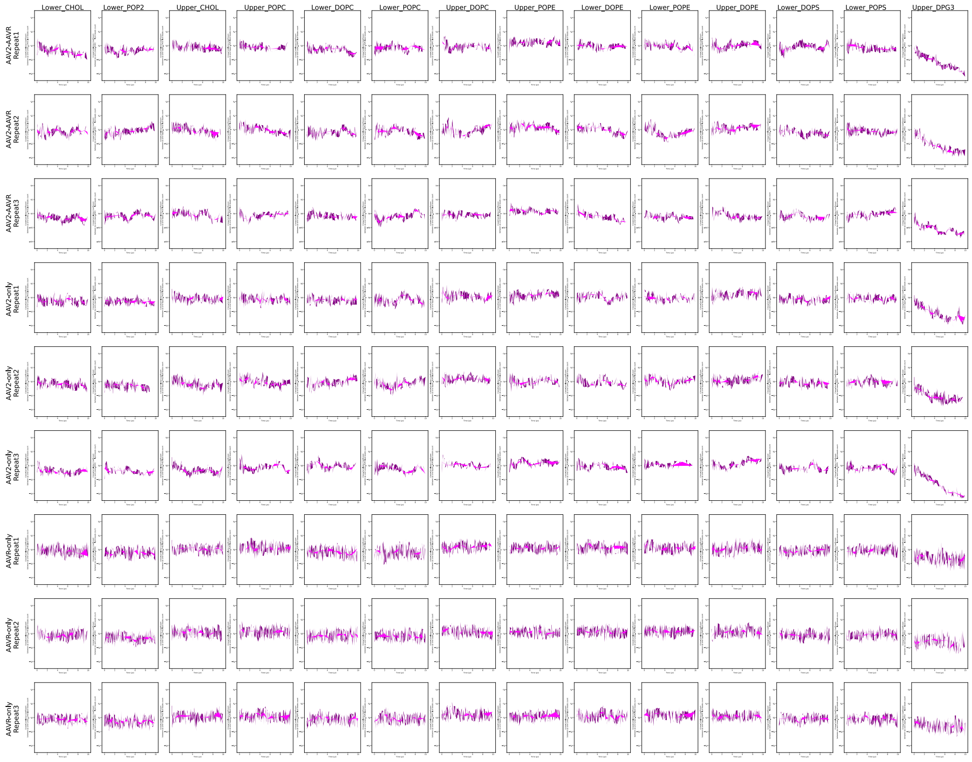

Figure S12 Cross-correlation results between the deviation from an average z-height (Δz) and the number of lipid species (L) for every lipid species and every simulation. **|** *The bright magenta colour represents the rolling average of window size 200 frames.*

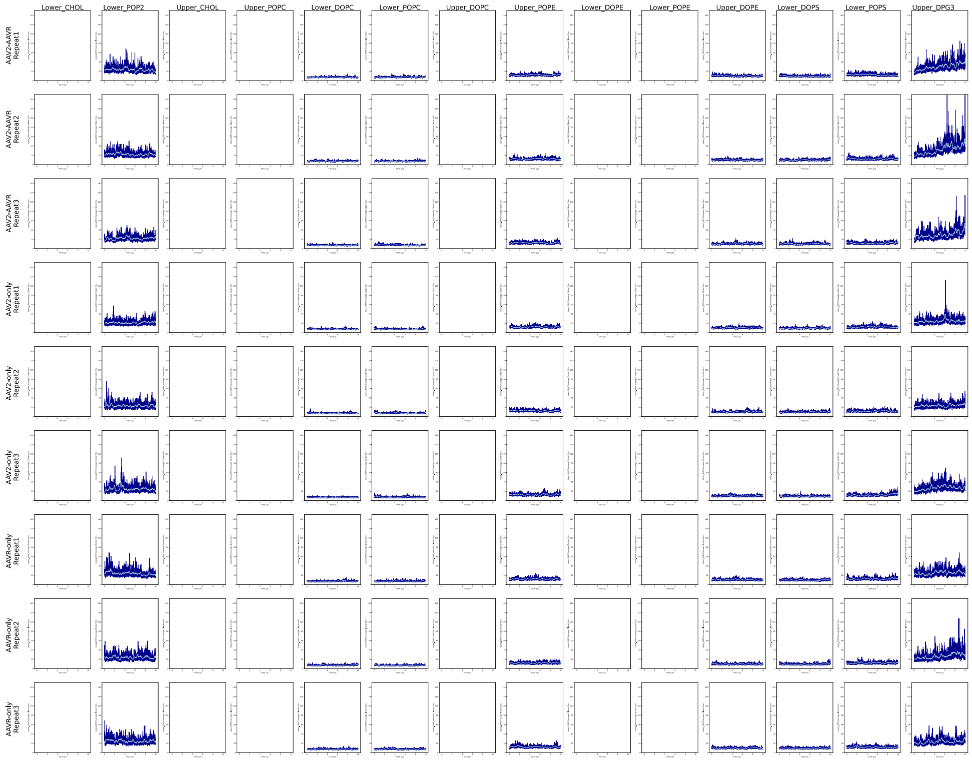

Figure S13 Cluster size for every lipid species and every simulation. **|** *The bright cyan colour represents the rolling average of the cluster size of 200 frames window size.*

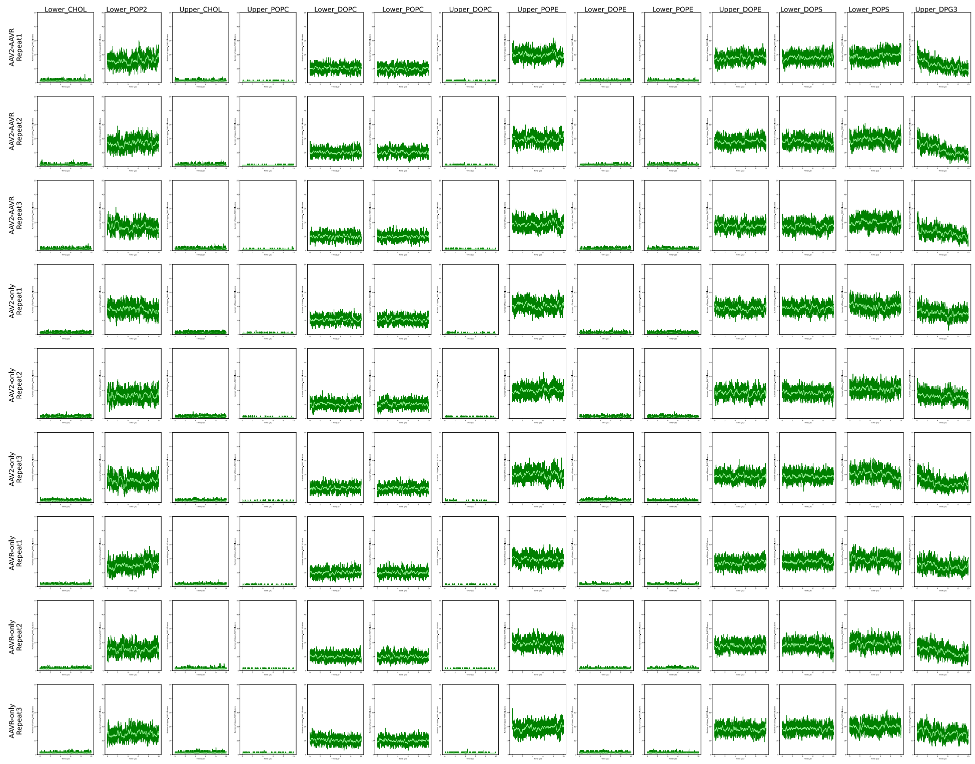

Figure S14 Number of clusters for every lipid species and every simulation. | *The bright cyan colour represents the rolling average of the cluster size of 200 frames window size.*

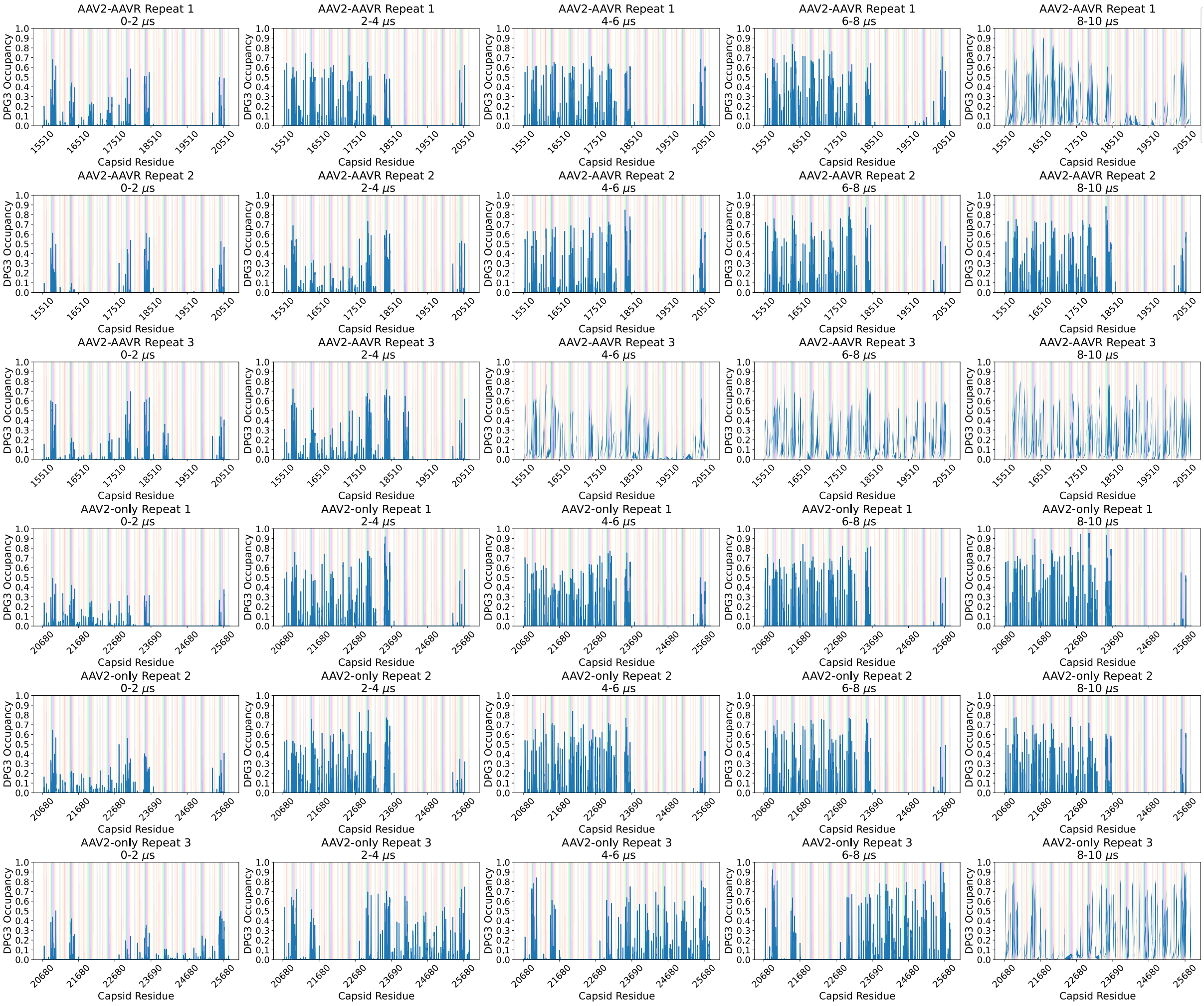

Figure S15 Occupancy plots of GM3 lipid on the membrane-binding pentamers in AAV2-AAVR system.

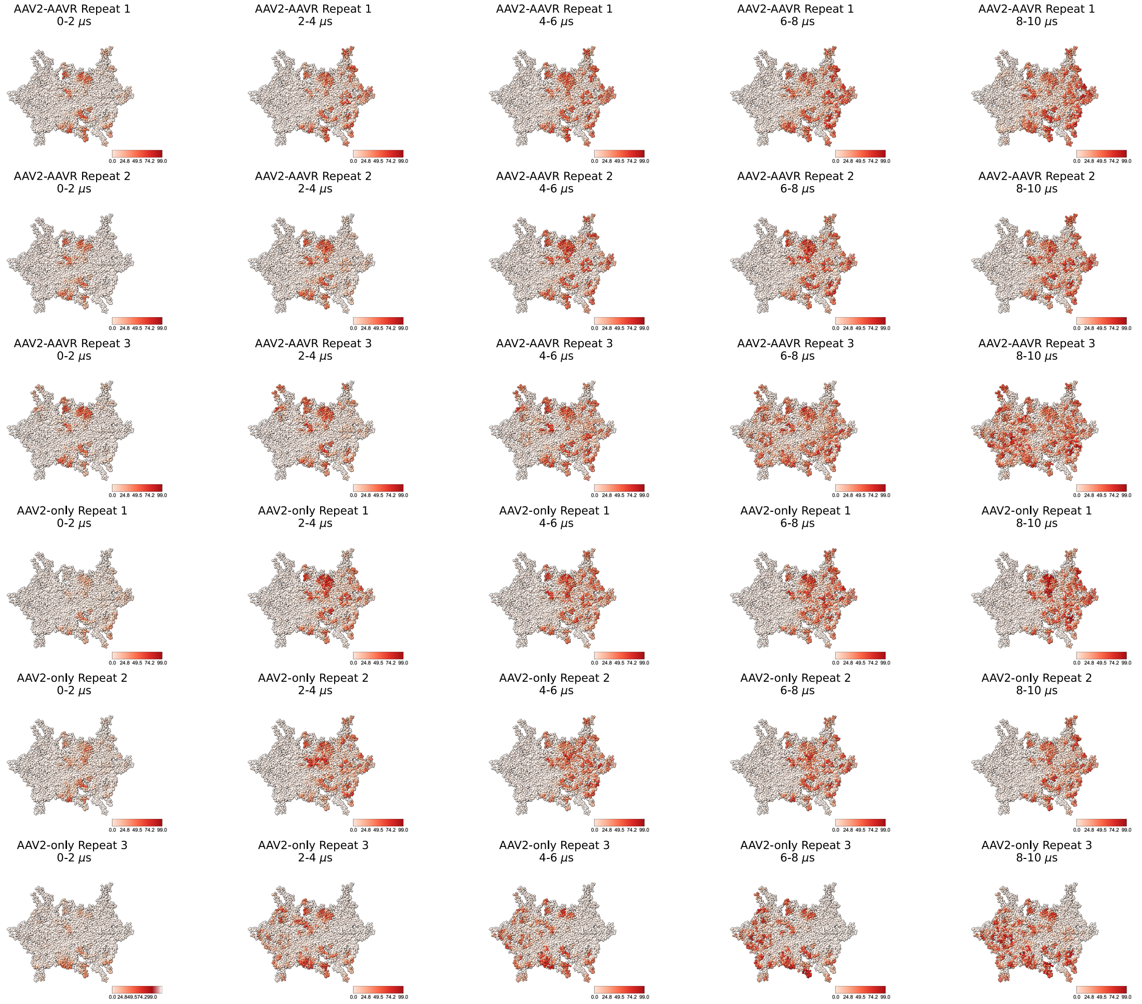

Figure S16 Occupancy diagrams of GM3 lipid on the membrane-binding pentamers in AAV2 system. **|** *The 2 pentamers are coloured by their occupancy within 2 μs with the highest intensity being the most occupied. 0.0 corresponds to 0.0 DPG3 beads occupancy. 99.0 corresponds to 1.0 DPG3 beads occupancy.*

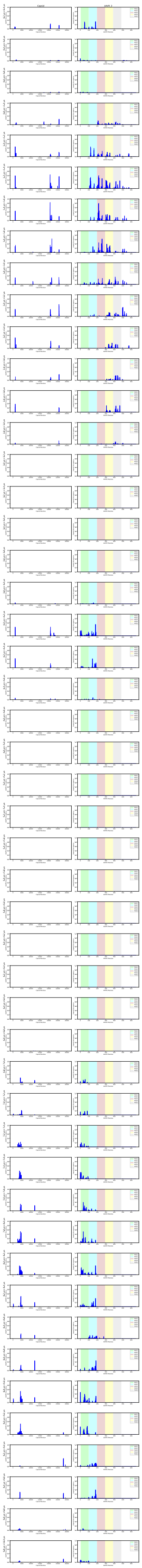

Figure S17 Occupancy of AAVR_3 on the membrane-binding pentamers in AAV2-AAVR system repeat 1.

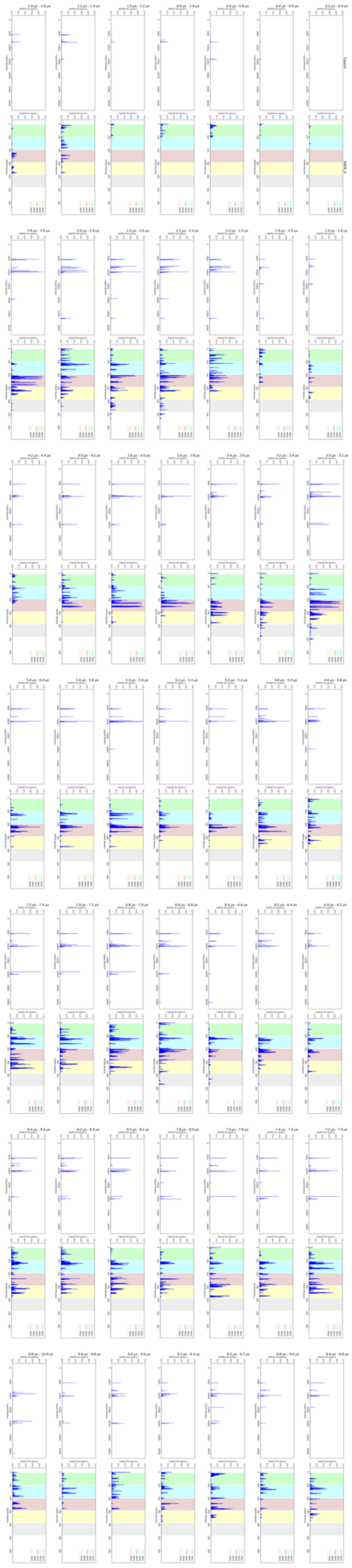

Figure S18 Occupancy of AAVR_4 on the membrane-binding pentamers in AAV2-AAVR system repeat 2.

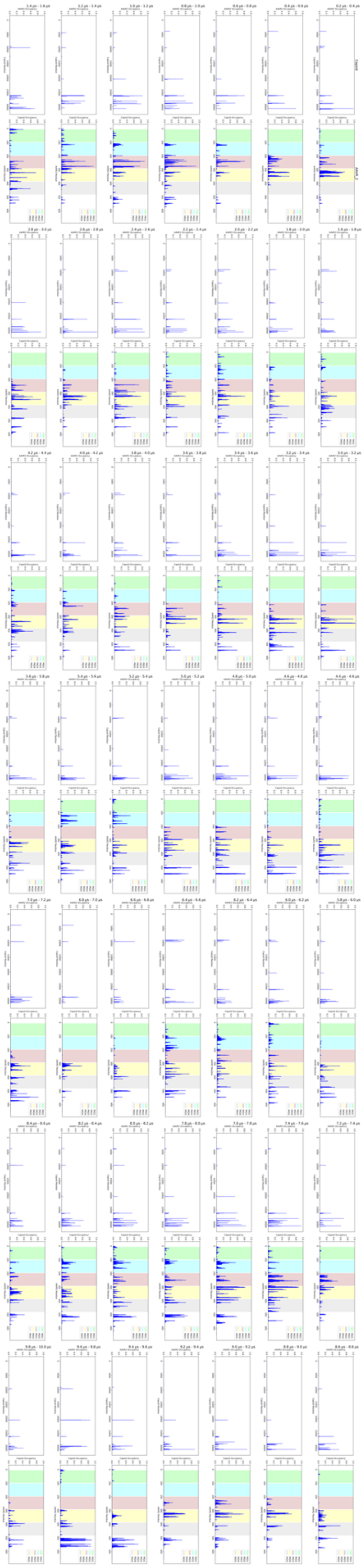

Figure S19 Occupancy of AAVR_2 on the membrane-binding pentamers in AAV2-AAVR system repeat 3.

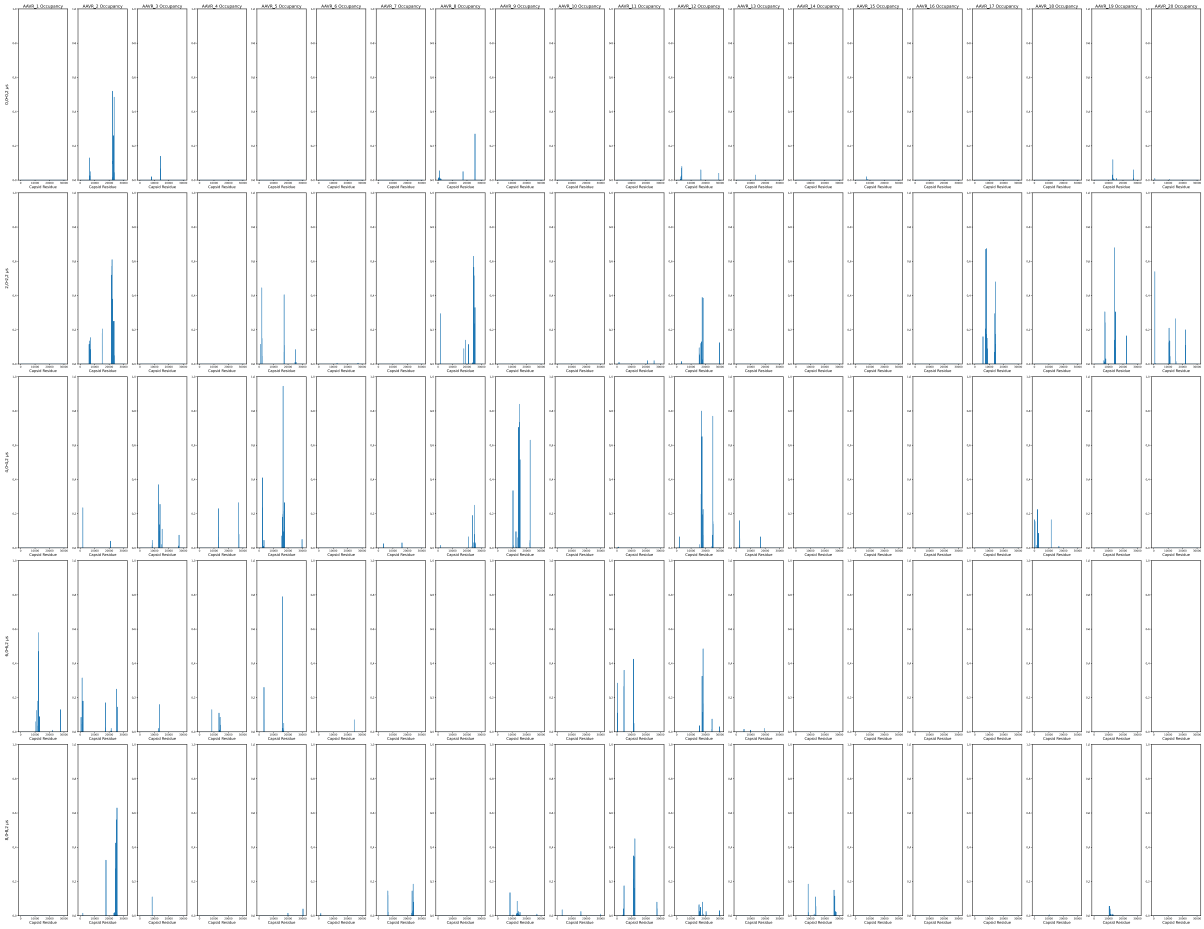

Figure S20 Occupancy of AAVR molecules on the capsid in the solution system repeat 1 sampled at every 2 μs.

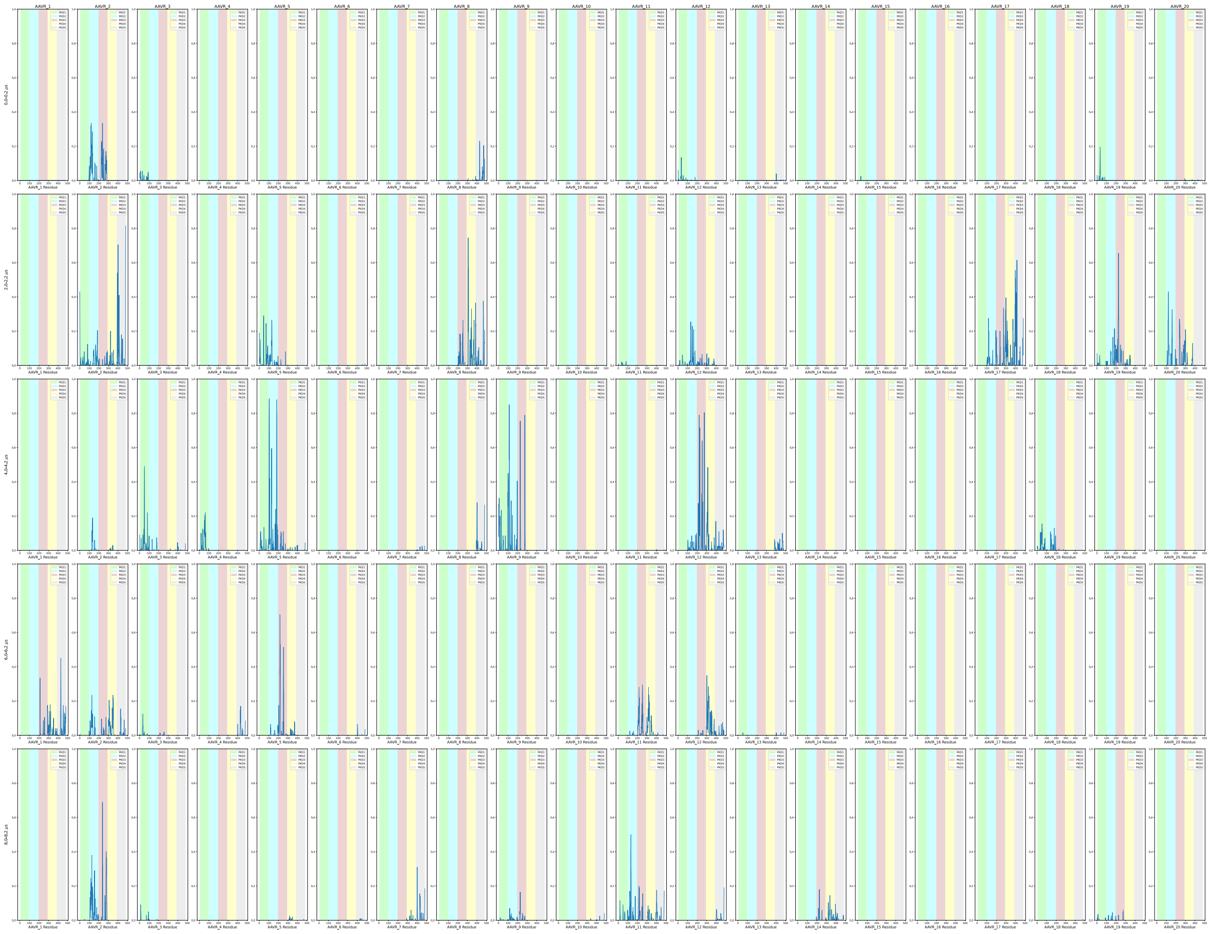

Figure S21 Occupancy of Capsid on the AAVR molecules in the solution system repeat 1 sampled at every 2 μs.

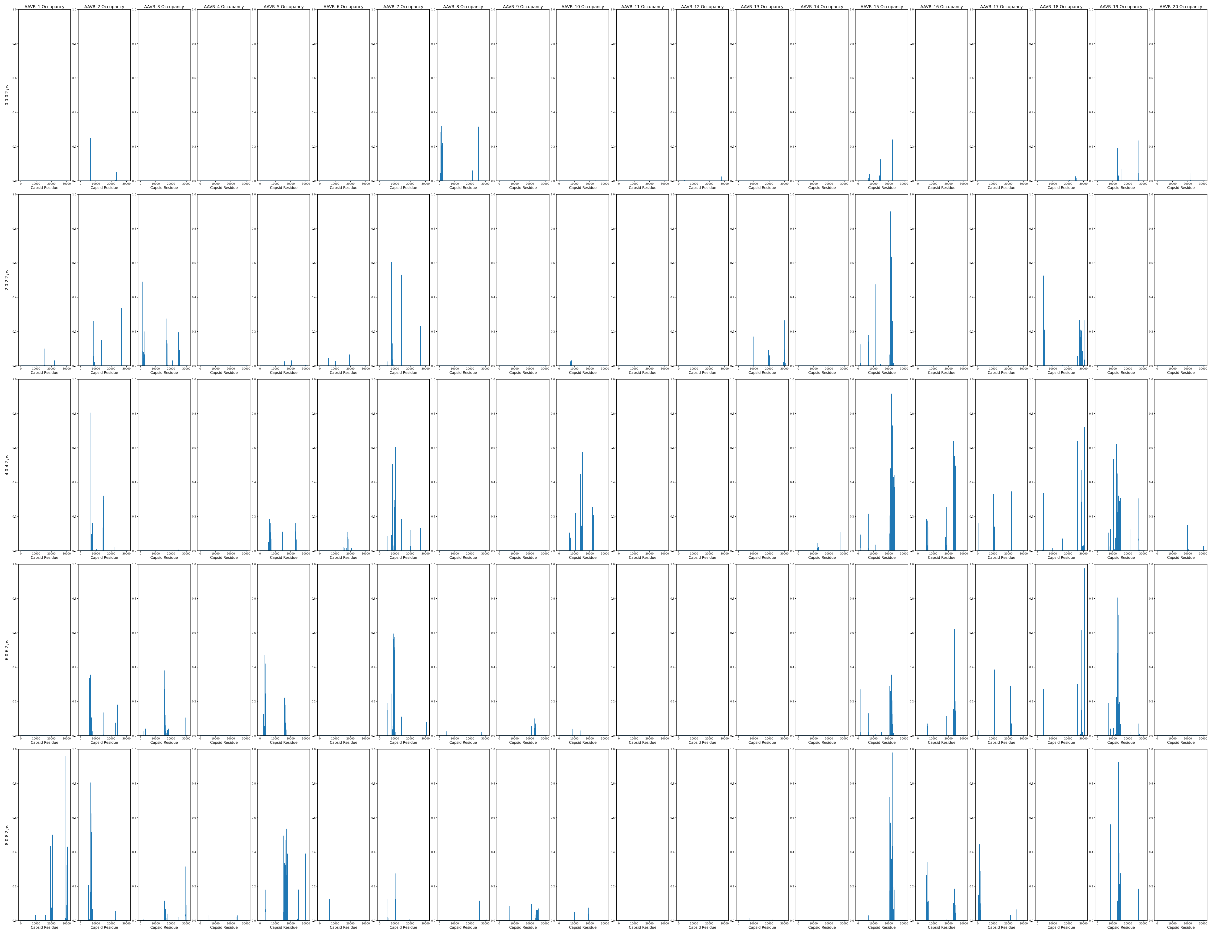

Figure S22 Occupancy of AAVR molecules on the capsid in the solution system repeat 2 sampled at every 2 μs.

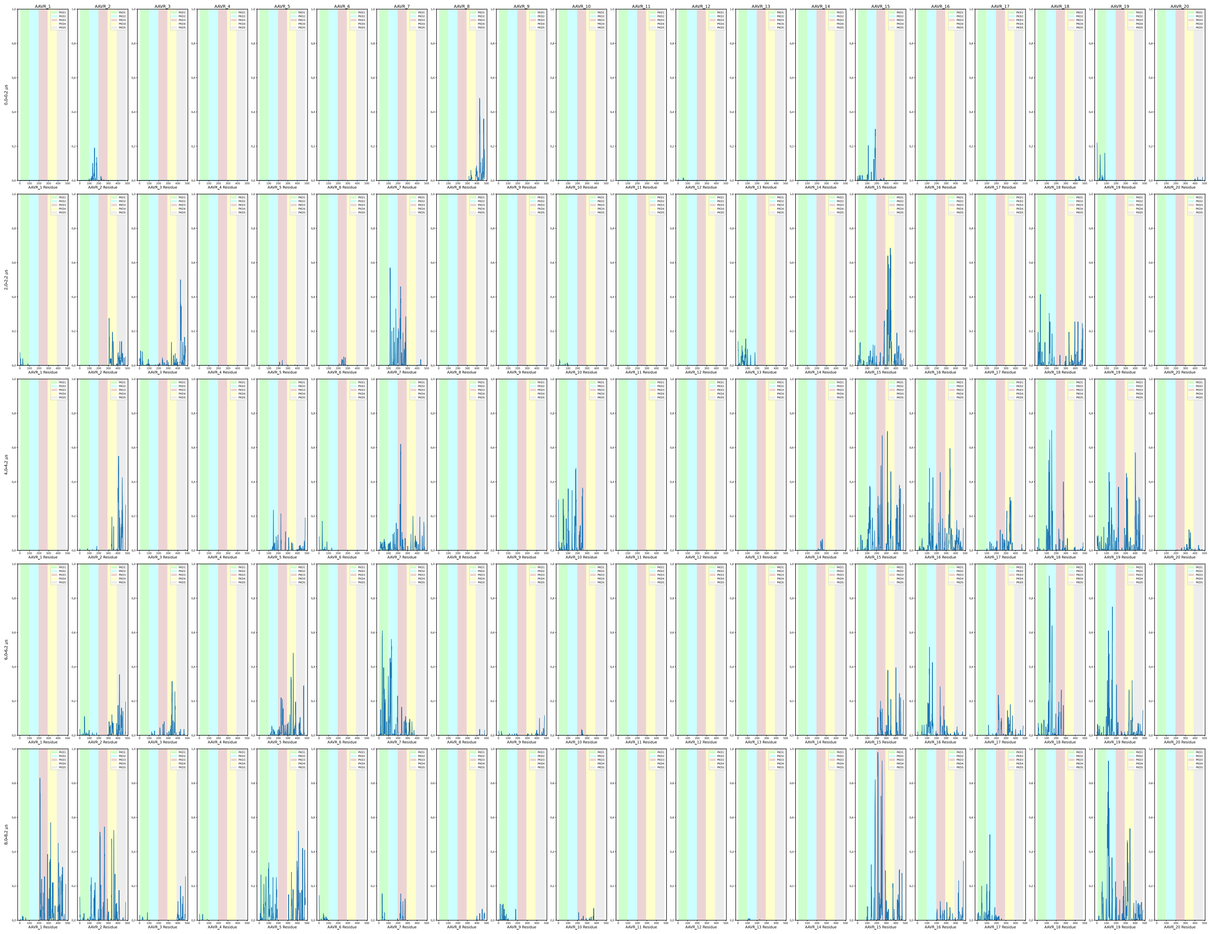

Figure S23 Occupancy of Capsid on the AAVR molecules in the solution system repeat 2 sampled at every 2 μs.

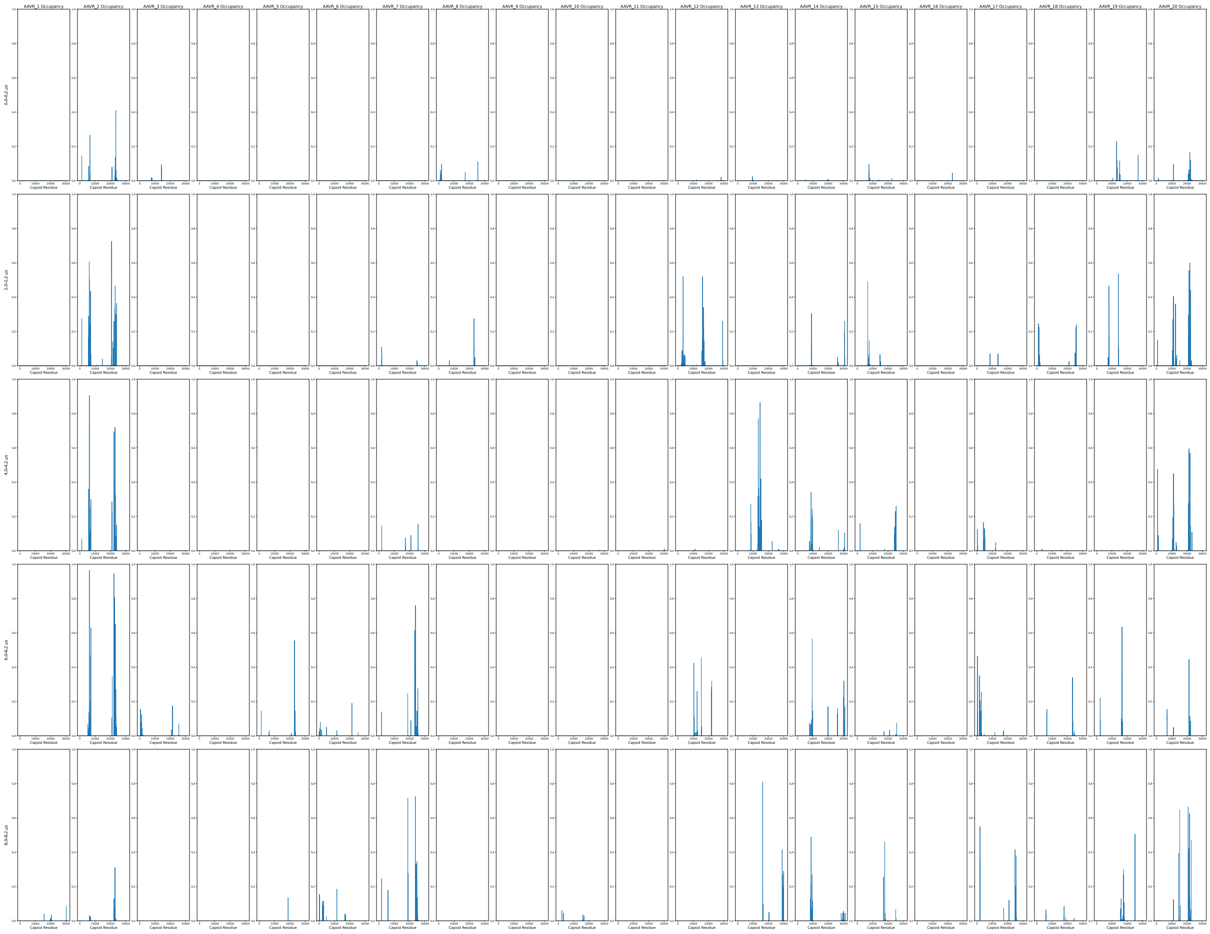

Figure S24 Occupancy of AAVR molecules on the capsid in the solution system repeat 3 sampled at every 2 μs.

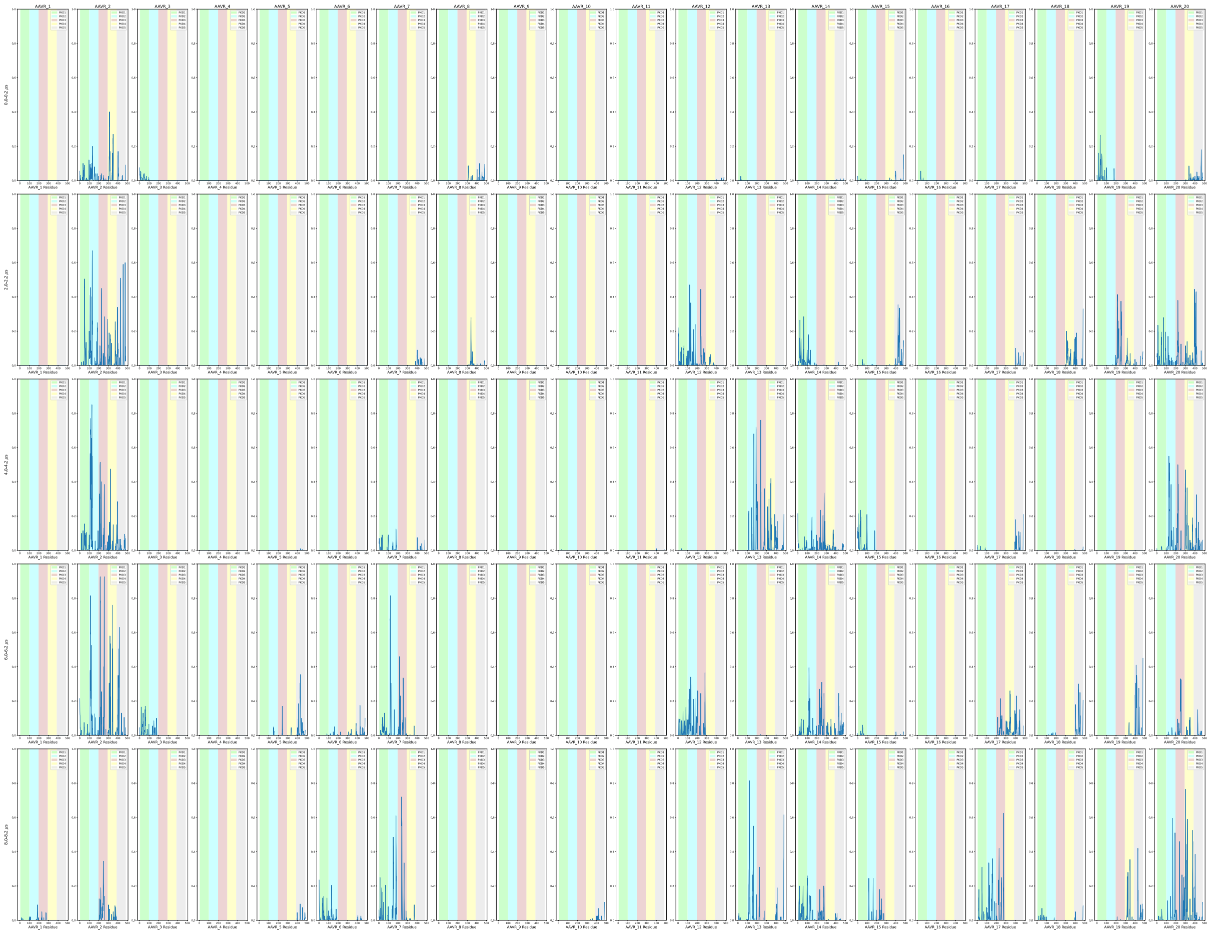

Figure S25 Occupancy of Capsid on the AAVR molecules in the solution system repeat 3 sampled at every 2 µs.

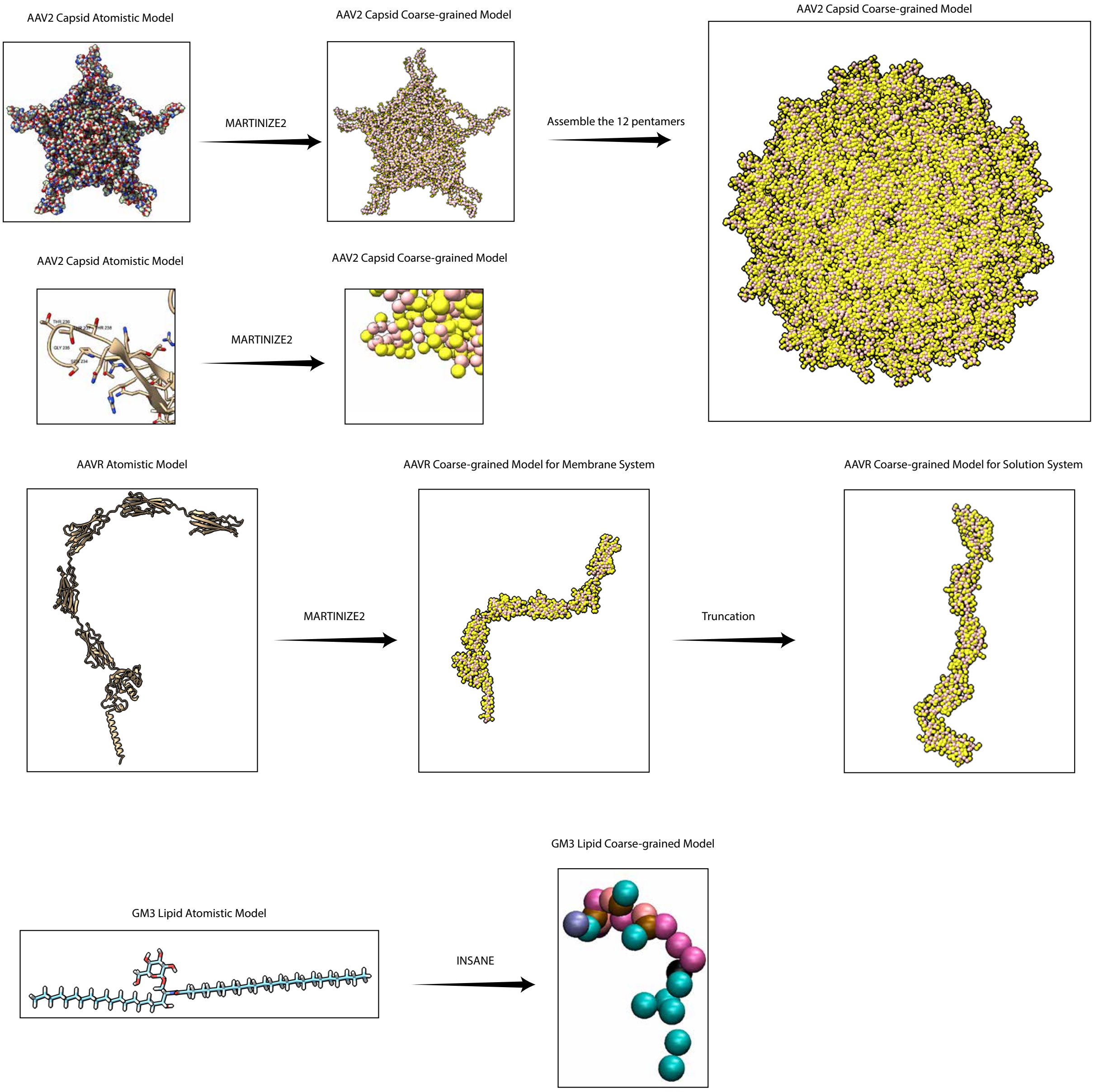

Figure S26 Diagram illustrating coarse-grained models building | *For proteins (capsid and AAVR), a pink coarse-grained bead is a backbone (BB) bead, a yellow coarse-grained bead is a sidechain (SC) bead. For GM3 lipid, colouring is based on the parametrisation by Grünewald F. et al. (2022). Parameters were generated from INSANE (Wassenaar T. et al., 2015) and MARTINIZE2 (Kroon P. C. et al., 2023).*

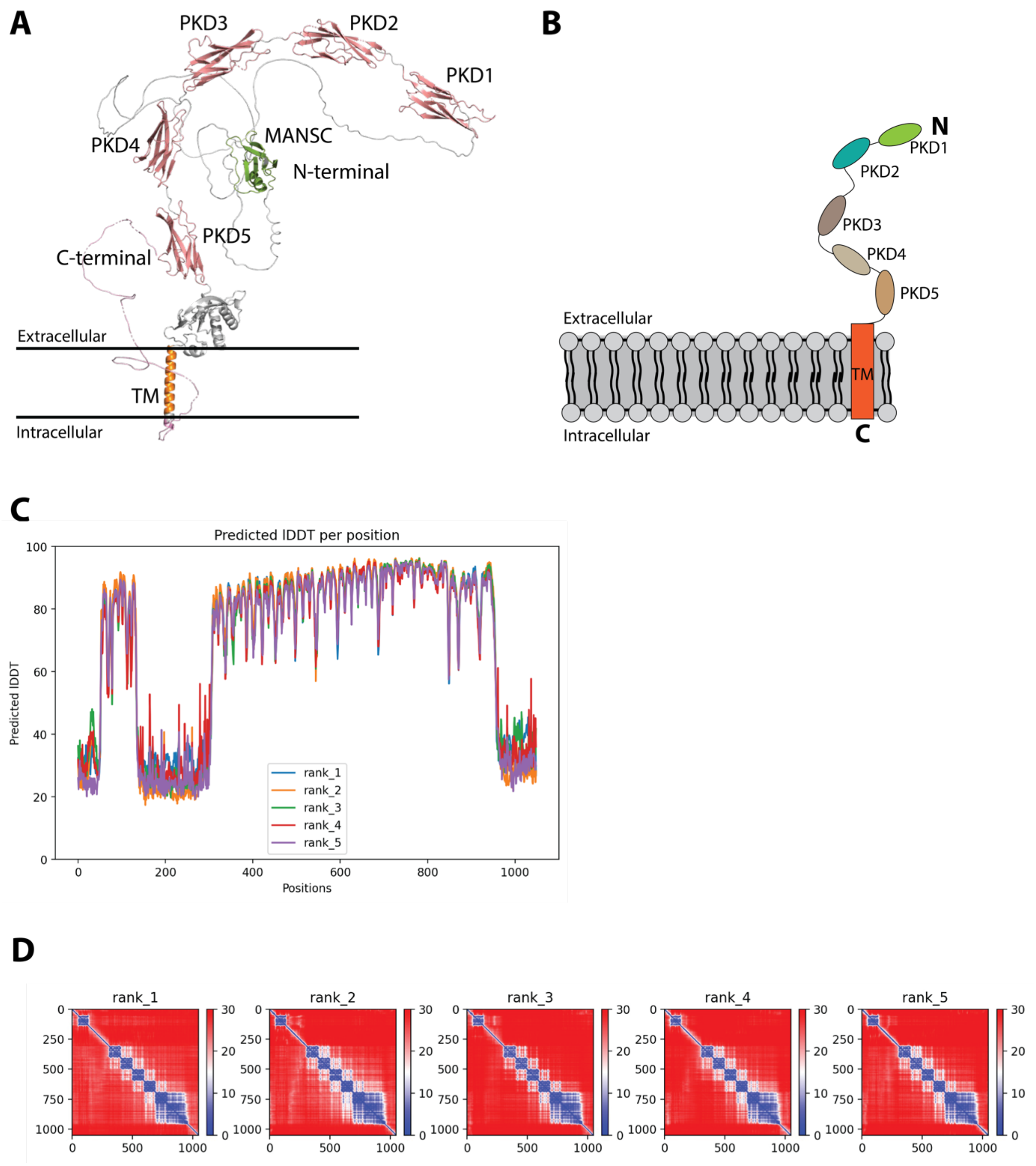

SI Figure 27 AlphaFold prediction results for the AAVR molecule* | *A *The output rank 1 prediction structure* | ***B*** *An illustration of the eventual structure used for coarse-grained modelling* | ***C*** *predicted LDDT score for all ranks. Notably, N-terminal domain including the MANSC domain (Residues 1 to 304) is largely formed by long strings of low confidence loops, hence this domain was not further included in the simulation as illustrated in* ***B****. Likewise, the C-terminal domain (Residues 961 onwards) was also removed before coarse-graining.* | ***D*** *Position Alignment Errors plots for all ranks showing good intra-domain packing.*

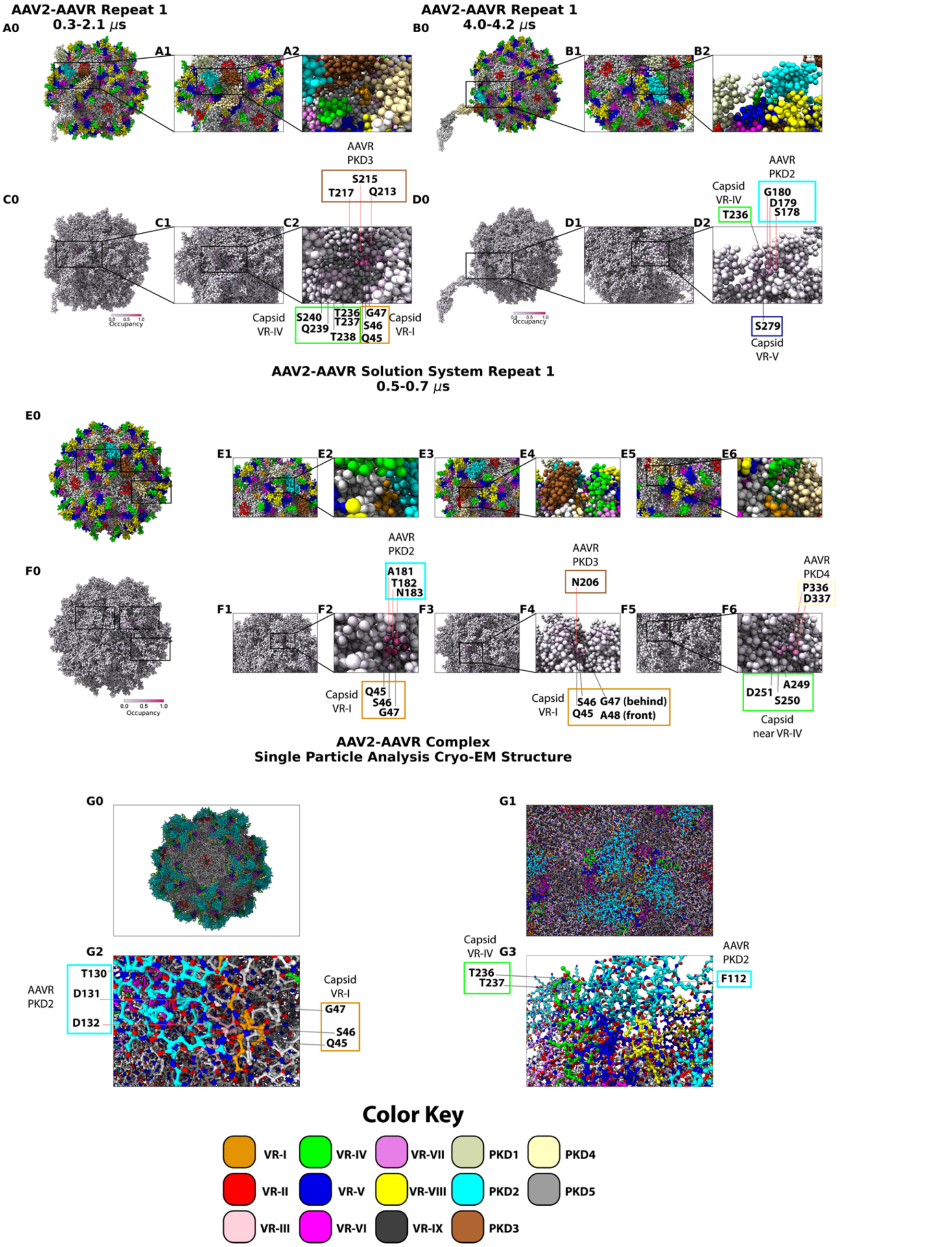

Figure S28 A representative subset of AAV2-AAVR complex structures captured from AAV2-AAVR membrane system simulations, AAV2-AAVR solution system simulations, and cryo-EM | ***A0-A2*** *are the AAV2-AAVR bound complex captured at 1.3 μs from AAV2-AAVR membrane system repeat 1.* | ***B0-B2*** *are the AAV2-AAVR bound complex captured at 4.1 μs from AAV2-AAVR membrane system repeat 1.* | ***C0-C2*** *are the AAV2-AAVR bound complex captured at 1.3 μs from AAV2-AAVR membrane system repeat 1 coloured by the occupancy level during 0.3 μs – 2.1 μs period.* | ***D0-D2*** *are the AAV2-AAVR bound complex captured at 4.1 μs from AAV2-AAVR membrane system repeat 1 coloured by the occupancy level during 4.0 μs – 4.2 μs period.* | ***E0-E6*** *are the AAV2-AAVR bound complex captured at 0.6 μs from AAV2-AAVR solution system repeat 1.* | ***F0-F6*** *are the AAV2-AAVR bound complex captured at 0.6 μs from AAV2-AAVR solution system repeat 1 coloured by the occupancy level during 0.5 μs – 0.7 μs period.* | ***G0-G3*** *are the representative binding sites based on the cryo-EM structure from Zhang R. et al. (2019a). PDB ID: 6IHB.*
